# Identification of the Novel Effector RsIA_NP8 in *Rhizoctonia solani* AG1 IA That Induces Cell Death and Triggers Defense Responses in Non-Host Plants

**DOI:** 10.3389/fmicb.2020.01115

**Published:** 2020-06-12

**Authors:** Miaomiao Wei, Aijun Wang, Yao Liu, Li Ma, Xianyu Niu, Aiping Zheng

**Affiliations:** ^1^State Key Laboratory of Crop Gene Exploration and Utilization in Southwest China, Sichuan Agricultural University, Chengdu, China; ^2^Rice Research Institute of Sichuan Agricultural University, Chengdu, China; ^3^Key Laboratory of Sichuan Crop Major Disease, Sichuan Agricultural University, Chengdu, China

**Keywords:** cell death, *Rhizoctonia solani* AG1 IA, signal peptide, N-glycosylation site, immune responses

## Abstract

*Rhizoctonia solani* AG1 IA is a necrotrophic fungus that causes rice sheath blight, one of the most significant rice diseases in the world. However, little is known about the pathogenic mechanisms and functions of effectors in *R. solani* AG1 IA. We performed functional studies on effectors in *R. solani* AG1 IA and found that, of 11 putative effectors tested, only RsIA_NP8 caused necrosis in the leaves of *Nicotiana benthamiana*. The predicted signal peptide of this protein was required to induce cell death, whereas predicted N-glycosylation sites were not required. RsIA_NP8 was upregulated during early infection, and the encoded protein was secreted. Furthermore, the ability of RsIA_NP8 to trigger cell death in *N. benthamiana* depended on suppressor of G2 allele of Skp1 (SGT1) and heat shock protein 90 (HSP90), but not on Mla12 resistance (RAR1) and somatic embryogenesis receptor-like kinase (SERK3). A natural variation that prevents the triggering of cell death in *N. benthamiana* was found in RsIA_NP8 in 25 *R. solani* AG1 IA strains. It is important to note that RsIA_NP8 induced the immune response in *N. benthamiana* leaves. Collectively, these results show that RsIA_NP8 is a possible effector that plays a key role in *R. solani* AG1 IA–host interactions.

## Introduction

The basidiomycete *Rhizoctonia solani* is a necrotrophic fungal pathogen that causes disease in many crops, such as rice, wheat, corn, cotton, and soybean. It contains 14 anastomosis groups (AG1–AG13 and AGBI) (Dölfors et al., 2019). *R. solani* does not produce asexual spores and can survive in the soil in sclerotial form (Li et al., 2019), which is a major cause of *R. solani* infestation. *R. solani* AG1 IA, the most destructive group of pathogens, causes rice sheath blight in rice-growing areas worldwide (Gautam et al., 2003; Lee and Rush, 1983). This pathogen mainly infects the leaf blade and sheath of rice plants, but all rice organs can be colonized by mycelia. Rice yield loss to *R. solani* AG1 IA has been recorded as high as 50% under favorable conditions (Bernardes-De-Assis et al., 2009). Despite these significant losses, little is known about the pathogenic mechanisms of *R. solani* AG1 IA.

Plant fungal pathogens are divided into biotrophs, hemibiotrophs, and necrotrophs based on lifestyle. Unlike biotrophs, which absorb nutrients from the cells and tissue of living hosts for colonization and growth, necrotrophs kill host cells and take nutrients from dead plant tissue (Schulmeyer and Yahr, 2017). However, all of these pathogens have a characteristic in common—effectors, which play crucial roles in promoting pathogen infection and suppressing host defenses (Koeck et al., 2011; Kabbage et al., 2015; Anderson et al., 2017). Although effectors are important, only a small proportion of the many proteins secreted by pathogens have been identified as effectors. SsCP1, an effector secreted by *Sclerotinia sclerotiorum*, interacts with pathogenesis-related protein 1 (PR-1) and plays an important role in successful host infection (Yang et al., 2018). The effectors SnToxA and SnTox3 recognize PR-1–PR-5 and PR-1-1, respectively, and play a crucial role in *Parastagonospora nodorum*–host interactions (Lu et al., 2014; Breen et al., 2016). Several effectors have been studied in species of *R. solani*, such as AGLIP1 (Li et al., 2019), RsLysM (Dölfors et al., 2019), and RSAG8_06778 (Anderson et al., 2017), in *R. solani* AG1 IA *R. solani* AG2-2IIIB, and *R. solani* AG8, respectively. AGLIP1 encodes a protein of 302 amino acids (aa) to trigger non-host and host cell death and affect the host immune response (Li et al., 2019).

Plant immune response depends on interactions between receptor proteins and effectors, and many nucleotide binding-leucine rich repeat (NBS-LRR) proteins have been identified as plant receptors (Stergiopoulos and de Wit, 2009). For example, the rice NBS-LRR protein–encoded gene Pi-ta interacts directly with the effector AvrPita in *Magnaporthe oryzae* to activate the immune response (Jia et al., 2000). Furthermore, the receptor-like proteins Cf-4, Cf-2, Cf-9, and Cf-4E in tomato interact with the effectors Avr4, Avr2, Avr9, and Avr4E in *Cladosporium fulvum*, respectively (Van der Hoorn et al., 2000; Luderer et al., 2002). In addition, recognition between receptor proteins and effectors leads to host cell death, which may promote necrotrophic pathogenesis (McDonald and Solomon, 2018). QCR8, a subunit of the cytochrome b-c1 complex of the mitochondrial respiratory chain, interacts with the effector SsSSVP1 in *S. sclerotiorum* to produce significant plant cell death, facilitating pathogen infection (Lyu et al., 2016).

According to genome sequencing, *R. solani* AG1 IA encodes 965 secreted proteins, some of which may be effectors (Zheng et al., 2013). However, only a few effector genes of *R. solani* AG1 IA have been functionally characterized and trigger defense signaling in plants. In this study, using transient expression assay, we detected the ability of 11 putative effectors in *R. solani* AG1 IA to induce cell death. The effector AG1 IA_05500 (named RsIA_NP8) triggered cell death in *Nicotiana benthamiana*. The predicted secretion signal peptide (SP) of this effector played a key role in inducing cell death. Furthermore, we found a natural variation in RsIA_NP8 that prevented the triggering of cell death in *N. benthamiana*. In addition, RsIA_NP8 triggered defense responses in *N. benthamiana*. These results facilitate understanding of molecular mechanisms behind host–*R. solani* AG1 IA interactions.

## Results

### RsIA_NP8 in *R. solani* Induces Cell Death in *N. benthamiana*

**FIGURE 1:**
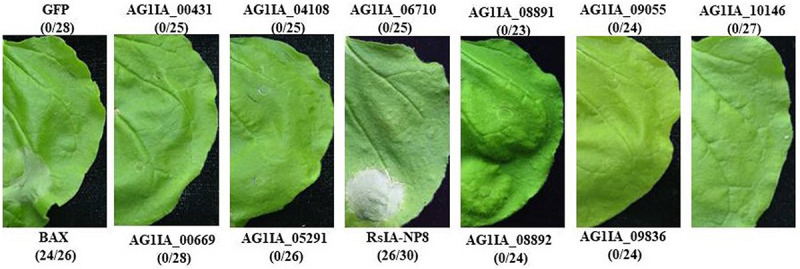
Putative effectors in *Rhizoctonia solani* AG1 IA induce cell death in *Nicotiana benthamiana* leaves. RsIA_NP8 induced cell death in *N. benthamiana*, whereas 10 other proteins did not. Green fluorescent protein (GFP) was used as a negative control. BAX was used as a positive control. Numbers e.g., 26/30, indicate that 26 of 30 infiltrated leaves exhibiting cell-death or mottling phenotypes. Representative photos were taken at 4 days post inoculation (dpi).

In previous studies, we obtained a 36.94 Mb draft genome sequence of *R. solani* AG1 IA and predicted 965 potential secreted proteins (Zheng et al., 2013). To detect potential novel effectors, we selected 11 genes for further testing, which are small secreted proteins (<310 aa), had a N-terminal SP, lacked a transmembrane domain, and were predicted to be effectors on http://effectorp.csiro.au/ (Sperschneider et al., 2018). The 11 putative effectors were cloned into 35S-PMDC32 expression vector to investigate their ability to induce cell death through agrobacterium-mediated transient expression in *N. benthamiana* leaves. Only AG1 IA-05500 (named RsIA_NP8) triggered cell death in *N. benthamiana* leaves 4 days after inoculation. However, a negative control of the green fluorescent protein (GFP) construct did not trigger cell death (Figure 1).

### RsIA_NP8 Is Highly Conserved in Different Fungi

**FIGURE 2:**
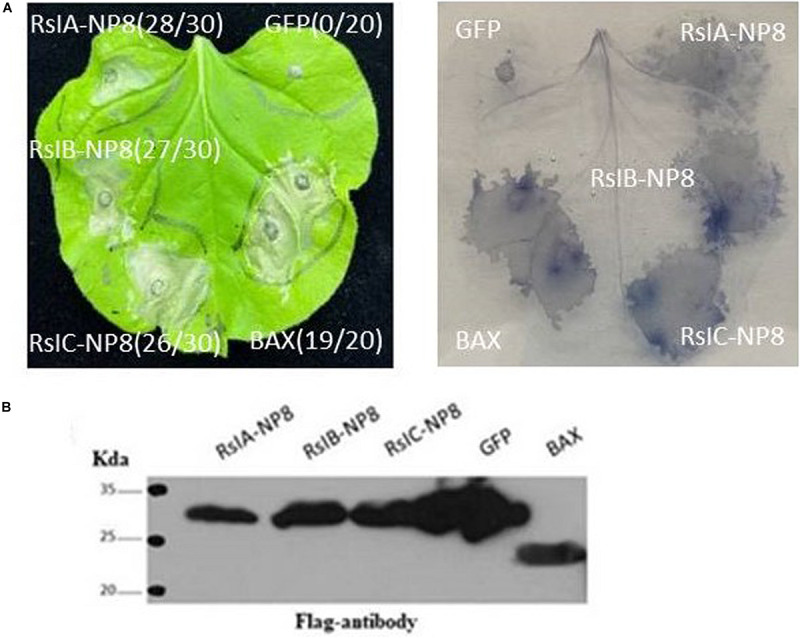
Homolog sequences of RsIA_NP8 from *R. solani* AG1 IB (RsIB_NP8) and *R. solani* AG1 IC (RsIC_NP8) induce cell death in *N. benthamiana* leaves. **(A)** RsIA_NP8, RsIB_NP8, and RsIC_NP8 induced cell death in *N. benthamiana*. Trypan blue staining of *N. benthamiana* leaves, GFP was used as a negative control. BAX was used as a positive control. Typical symptoms were photographed at four dpi. Numbers e.g., 28/30, indicate that 28 of 30 infiltrated leaves exhibiting cell-death or mottling phenotypes. **(B)** Transient expression of RsIA_NP8, RsIB_NP8, RsIC_NP8, BAX, and GFP in *N. benthamiana* was confirmed by Western blotting.

RsIA_NP8 encodes a 304-aa protein that contains a predicted N-terminal SP (22 aa) and two internal repeat motifs (RPT; 25–92 and 108–174 aa, respectively). In general, the effector proteins of plant pathogenic fungi are highly conserved (Lee and Rose, 2012). We found several homolog genes of RsIA_NP8 in plant pathogenic fungi that contained *R. solani* AG1 IB, *R. solani* AG1 IC, and *R. solani* AG3 (Supplementary Figure S1). Furthermore, we obtained homolog sequences of RsIA-NP8 from *R. solani* AG1 IB (RsIB_NP8) and *R. solani* AG1 IC (RsIC_NP8) by polymerase chain reaction (PCR) using primers designed based on *R. solani* AG1 IB and *R. solani* AG1 IC (Supplementary Table S1). Both RsIB_NP8 and RsIC_NP8 triggered cell death in *N. benthamiana* leaves (Figure 2A). Western blotting showed that RsIB_NP8 and RsIC_NP8 were expressed in the infiltrated *N. benthamiana* leaves (Figure 2B). These results show that homologs of RsIA_NP8 are widely present in different plant pathogenic fungi, in particular three *R. solani* AG1 fungi.

### Functional Validation of the Predicted SP of RsIA_NP8

**FIGURE 3:**
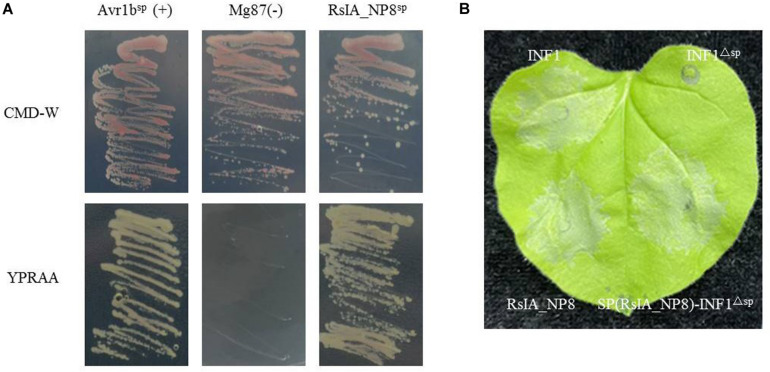
The signal peptide (SP) of RsIA_NP8 is functional. **(A)** Functional validation of the SP of RsIA_NP8 using yeast invertase secretion assay. All transformed YTK12 yeast strains grew on YPRAA media with raffinose as the sole carbon source (1% yeast extract, 2% peptone, 2% raffinose, and 2 μg antimycin A per liter). N-terminal sequences of *Phytophthora sojae* Avr1b and *Magnaporthe oryzae* Mg87 were used as positive and negative controls, respectively. The untransformed YTK12 did not grow on either CMD-W (0.67% yeast N base without amino acids, 0.075% tryptophan dropout supplement, 2% sucrose, 0.1% glucose, and 2% agar) or YPRAA media. Yeast growth on CMD-W media was equally viable among the transformed strains. Mg87: negative control Mg87 SPs; Avr1b^SP^: positive control Avr1b SPs; RsIA_NP8^SP^: SPs of RsIA_NP8. **(B)** Functional validation of the SP of RsIA_NP8 by experiments of swap the RsIA_NP8^SP^ with a SP of INF1.

A yeast secretion assay was used to identify the predicted SP of RsIA_NP8 (Lee and Rose, 2012). The predicted SP nucleotide sequence of RsIA_NP8 was fused in frame with the truncated pSUC2 gene, which encodes invertase lacking its own SP. The fusion vector was transformed into yeast strain YTK12, which is deficient in invertase secretion. Invertase containing functional SP degrades raffinose into simple sugars, and thus YTK12 will grow on media with raffinose as a carbon source (Oh et al., 2009; Tian et al., 2011). YTK12 strains with the predicted SP of RsIA_NP8 or the secretion signal of *Phytophthora sojae* Avr1b (positive control) grew on the raffinose-containing YPRAA (Figure 3A). However, as a negative control, the N-terminus of Mg87 in *M. oryzae* did not grow on YPRAA medium (Figure 3A; Gu et al., 2011). Additionally, when the SP of INF1 elicitin was substituted by that of RsIA_NP8, the fusion protein could successfully induce cell death in *N. benthamiana* (Figure 3B). These results indicate that the predicted SP of RsIA_NP8 leads to the secretion of invertase and functionally secreted proteins.

### RsIA_NP8 Expression During *R. solani* AG1 IA Infection

**FIGURE 4:**
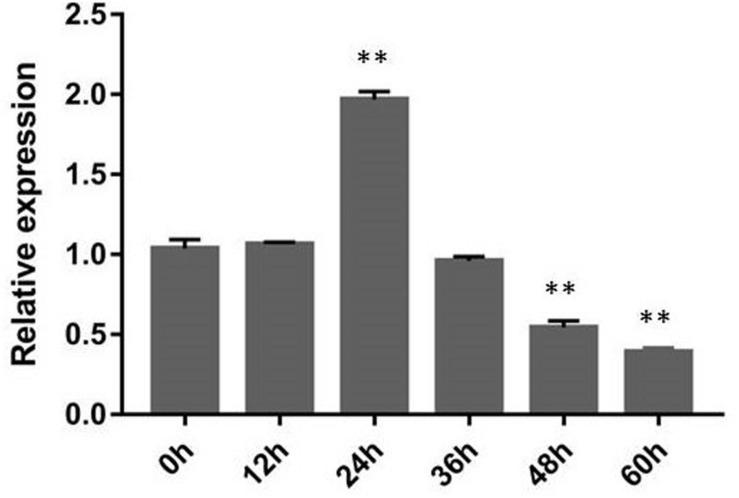
Expression of RsIA_NP8 during *R. solani* AG1 IA infection of the sheath blight–susceptible rice cultivar 9311. Rice sheaths inoculated against *R. solani* AG1 IA were collected 0, 12, 24, 36, 48, and 60 h after inoculation for gene expression analyses using quantitative real-time reverse transcription polymerase chain reaction. 18S rRNA expression was used as an internal reference for normalizing within the samples. Error bars indicated the standard deviation of four independent replicates (^∗∗^*P* < 0.01).

Fungal effector genes are generally induced by transcription in invading plant cells (Stergiopoulos and de Wit, 2009). RsIA_NP8 expression is upregulated at the 24-h inoculation point (hpi) according to transcriptome data (Zheng et al., 2013). To detect change in RsIA_NP8 expression during *R. solani* AG1 IA infection, we studied RsIA_NP8 expression in rice cultivar 9311, which is susceptible to *R. solani* AG1 IA. RsIA_NP8 expression at the 12, 24, 36, 48, and 60 hpi was identified by quantitative real-time reverse transcription (qRT) PCR. RsIA_NP8 was upregulated at 24 hpi, consistent with transcriptome data (Figure 4). These findings indicate that RsIA_NP8 is upregulated during the early stage of AG1 IA infection and plays crucial roles in *R. solani* AG1 IA–rice interactions.

### The Predicted SP and Motifs of RsIA_NP8 Are Required to Induce Cell Death

**FIGURE 5:**
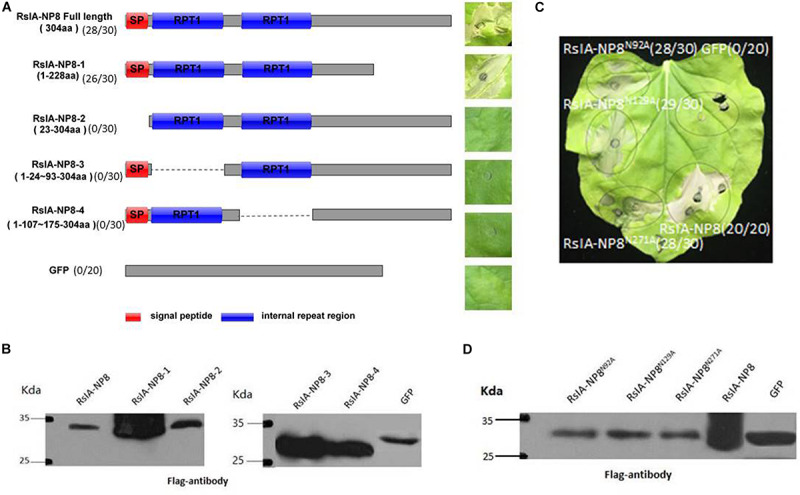
The predicted motifs and signal peptide of RsIA_NP8 are required to induce cell death. **(A)** Expression of RsIA_NP8 and its mutants in *N. benthamiana* by agro-infiltration. Typical symptoms were photographed at four dpi. Numbers e.g., 28/30, indicate that 28 of 30 infiltrated leaves exhibiting cell-death or mottling phenotypes. **(B)** Expression of mutant proteins in infiltrated leaves detected by Western blotting. **(C)** Functional characterization of three putative N-glycosylation sites for RsIA_NP8. Typical symptoms were photographed at four dpi. Numbers e.g., 28/30, indicate that 28 of 30 infiltrated leaves exhibiting cell-death or mottling phenotypes. **(D)** Expression of three putative N-glycosylation site mutant proteins in infiltrated leaves detected by Western blotting.

The SPs of multiple pathogenic fungi effectors are required to induce cell death in plants (Saitoh et al., 2012). To identify the function of the predicted SP of RsIA_NP8 in inducing cell death, we performed transient expression assays of RsIA_NP8 with and without the SP in *N. benthamiana* leaves. RsIA_NP8^Δ^ sp (RsIA_NP8 lacking the SP) no longer induced cell death (Figure 5A). Western blotting showed that RsIA_NP8^Δ^ sp was expressed in the infiltrated leaves (Figure 5B). These results show that the SP is required for RsIA_NP8 to trigger cell death in *N. benthamiana.* Furthermore, to clarify the function of the two predicted RPT motifs of RsIA_NP8 in inducing cell death, we analyzed deletion mutants of RsIA_NP8 in *N. benthamiana* using agroinfiltration. The lack of any one RPT motif did not lead to cell death (Figure 5A). Immunoblot analyses showed that all deletion mutants of RsIA_NP8 were expressed in *N. benthamiana* leaves (Figure 5B). These data indicate that the two RPT motifs are required to induce cell death.

Some effector proteins of filamentous fungal pathogens are predicted to be N-glycosylated, such as effector Slp1 in *M. oryzae*, which causes rice blast (Chen et al., 2014). In addition, three putative N-glycosylation sites (Asn-92, Asn-129, and Asn-271) are predicted in the RsIA_NP8 sequence. To determine whether these N-glycosylation sites are necessary for RsIA_NP8 to induce cell death, we replaced them with alanine. Cell death in *N. benthamiana* leaves was detected 4 days after infiltration of an agrobacterium containing the RsIA_NP8 mutation (Figure 5C). The expression of RsIA_NP8 truncated variant (Asn-92, Asn-129, and Asn-271) in *N. benthamiana* was verified by Western blotting (Figure 5D). These findings indicate that the predicted N-glycosylation sites do not affect the ability of RsIA_NP8 to induce cell death.

### RsIA_NP8-Triggered Cell Death in *N. benthamiana* Depends on SGT1 and HSP90 but Not RAR1 and SERK3/Bak1

**FIGURE 6:**
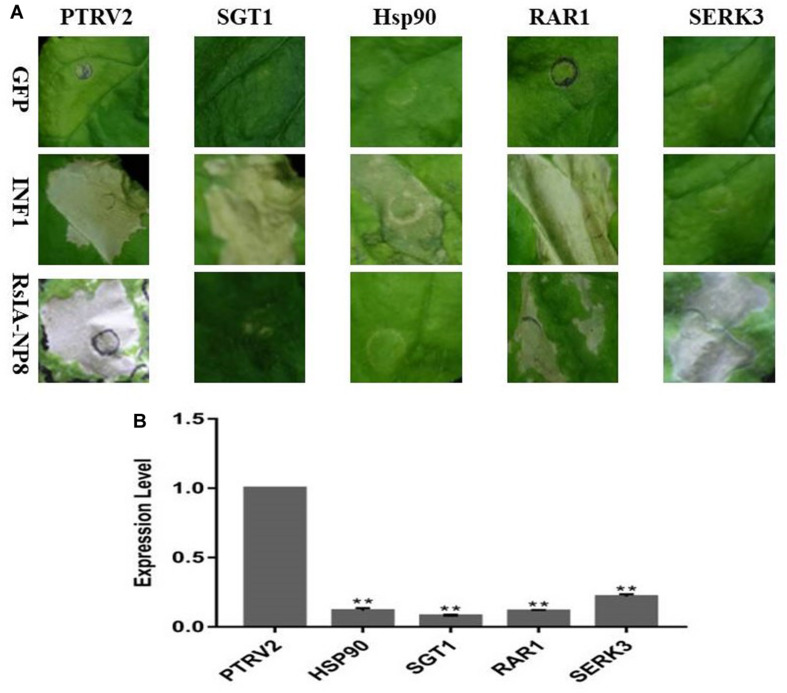
SGT1 and Hsp90 are required for RsIA_NP8-induced cell death in *N. benthamiana*. **(A)** RsIA_NP8 was transiently expressed in *N. benthamiana* leaves silenced for pTV00 (control), SGT1, Hsp90, RAR1, and SERK3. GFP and INF1 were used as control proteins. Typical symptoms were photographed 4 days after agro-infiltration. The experiment was repeated three times with similar results. **(B)** Transcription of genes in silenced *N. benthamiana* measured by quantitative RT-PCR. Error bars represent standard errors from three biological replicates (**P < 0.01).

Suppressor of G2 allele of Skp1 (SGT1), heat shock protein 90 (HSP90), and Mla12 resistance (RAR1) play crucial roles in resistance induced by R protein (Shirasu and SchulzeLefert, 2003; Shirasu, 2008). Therefore, we performed a VIGS assay against these genes in *N. benthamiana* to identify the function of SGT1, HSP90, and RAR1 in cell death induced by RsIA_NP8; INF1 was used as a positive control. RsIA_NP8 induced cell death in RAR1-silenced plants but not in SGT1- and HSP90-silenced plants (Figure 6A). The expression of these genes was verified by RT–quantitative PCR and was markedly reduced in silenced plants compared to the control (Figure 6B). These results indicate that RsIA_NP8-triggered cell death depends on SGT1 and HSP90 but not RAR1. In addition, somatic embryogenesis receptor-like kinase two (SERK3)/Bak1, which encodes receptor-like kinase, is a key regulator in pathogen-associated molecular pattern (PAMP), such as programmed cell death by INF1 (Heese et al., 2007). To further determine whether RsIA_NP8 is involved in PAMP-triggered immunity (PTI) responses, we next tested whether SERK3/Bak1 mediates RsIA_NP8-induced cell death in *N. benthamiana*. The results showed that RsIA_NP8 induced cell death in SERK3/Bak1-silenced plants. Therefore, RsIA_NP8 is not involved in PTI responses dependent on SERK3/Bak1 in *N. benthamiana*.

### Natural Variation Prevents RsIA_NP8 From Triggering Cell Death

**FIGURE 7:**
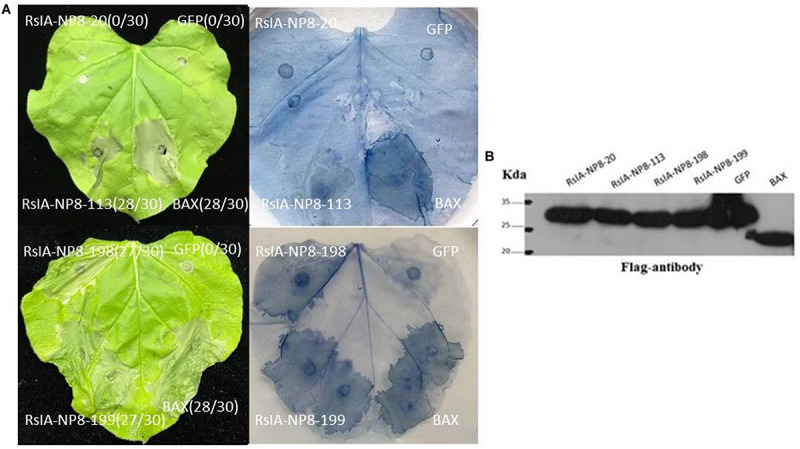
Natural variation prevents RsIA_NP8 from triggering cell death. **(A)** Expression of RsIA_NP8 mutant proteins in *N. benthamiana* by agro-infiltration. Typical symptoms were photographed 4 days after agro-infiltration. Numbers e.g., 0/30, indicate that 0 of 30 infiltrated leaves exhibiting cell-death or mottling phenotypes. **(B)** Expression of mutant proteins in infiltrated leaves detected by Western blotting.

We further analyzed sequences of RsIA_NP8 in 25 *R. solani* AG1 IA strains isolated from different areas in China. Among the 25 strains, four natural variations in RsIA_NP8 were identified with PCR (Supplementary Figure S2). To determine the ability of these variations to induce cell death, we performed transient expression assays of the RsIA-NP8 variations in *N. benthamiana* leaves. RsIA_NP8-20 (variation found in 20 aa) no longer induced cell death (Figure 7A); however, cell death was induced in *N. benthamiana* leaves by the other variations (Figure 7A). Western blotting showed that RsIA_NP8-20 was expressed in the infiltrated leaves (Figure 7B). These results show that this natural variation prevents RsIA_NP8 from triggering cell death in *N. benthamiana*.

### Subcellular Localization of RsIA_NP8

**FIGURE 8:**
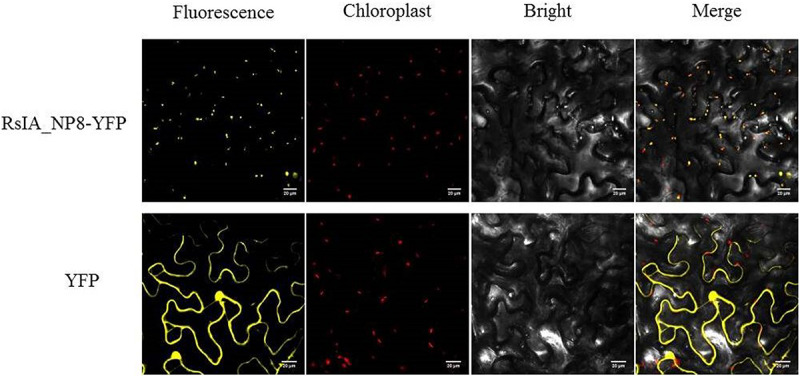
Subcellular localization of RsIA_NP8 transiently expressed in *N. benthamiana* leaves. The vector PHB carrying YEP was used as a control. Bars = 20 μm.

To explore the subcellular localization of RsIA_NP8, we transiently expressed 2 × 35S:RsIA_NP8-YFP in tobacco leaf epidermis cells. Transient expression of 2 × 35S:YFP was used as a control. The RsIA_NP8-YFP protein was localized predominantly in the chloroplasts of the transiently transformed tobacco leaf epidermis cells compared to the control (Figure 8).

### RsIA_NP8 Triggers Plant Immunity Responses

**FIGURE 9:**
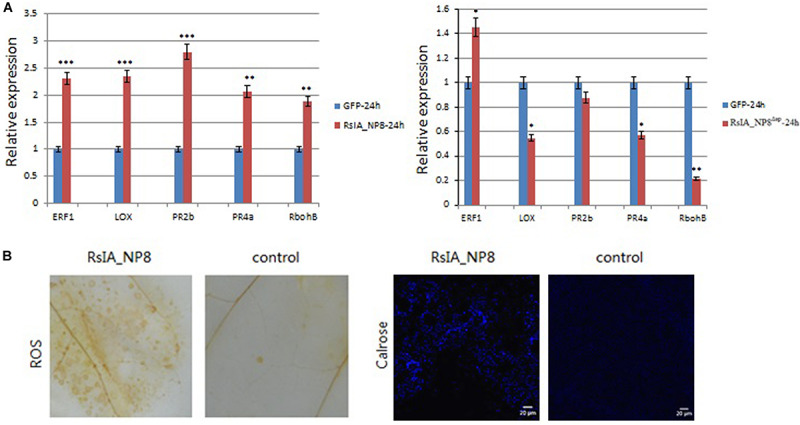
RsIA_NP8 triggers plant immunity responses in *N. benthamiana*. **(A)** Expression of genes related to plant immunity in *N. benthamiana* leaves transiently expressing RsIA_NP8 and RsIA_NP8 with SP deletion mutant for 24 h. Error bars indicated the standard deviation of four independent replicates (*P < 0.05; **P < 0.01; ***P < 0.001). **(B)** Accumulation of reactive oxygen species (ROS) and deposition of callose in *N. benthamiana*. For observation of callose, Bars, 20 μm. These experiments were replicated three times with six leaves per biological replicate.

To identify whether RsIA_NP8 activates the immune response in *N. benthamiana*, we used qRT-PCR to evaluate the expression of several genes related to activation of the immune response in *N. benthamiana.* Transcription factor ERF1 (ethylene response factor 1), LOX (lipoxygenase), RbohB (respiratory burst oxidase homolog protein B), and the two pathogenesis-related (PR) protein genes PR2b and PR4a were strongly induced by RsIA_NP8 at 24 h (Figure 9A; Wang et al., 2000; Lorenzo et al., 2003; Lee et al., 2013); however, the expression of these five genes did not strongly induce upregulation by RsIA_NP8 with SP deletion mutant at 24 h (Figure 9A). These results indicate that RsIA_NP8 has an important role in activating early defense responses in hosts. In addition, we investigate the ability of RsIA_NP8 to activate hydrogen peroxide (H_2_O_2_) and callose deposition in *N. benthamiana* leaves. It was shown that H_2_O_2_ was activated by RsIA_NP8 at 4 dpi compared to the control, and extensive callose deposition was detected at 24 h after infiltration *N. benthamiana* leaves (Figure 9B).

## Discussion

Effectors play a crucial role in the successful infection of host plants by pathogens (Jones and Dangl, 2006; Stergiopoulos and de Wit, 2009). In recent years, hundreds of effectors have been predicted in various plant pathogenic fungi through genome sequencing and RNA-seq (Cantu et al., 2013; Hane et al., 2014). In the *R. solani* AG1 IA genome, 965 genes encode secreted proteins, of which 234 show significantly different expression during the early infection process, and 103 small cysteine-rich proteins are predicted to be effectors (Zheng et al., 2013). Li et al., 2019 reported that the effector AGLIP1 in *R. solani* AG1 IA induces cell death in *N. benthamiana* and rice protoplasts.

In this study, we selected 11 candidate effectors for functional assay; of these, only RsIA_NP8 triggered cell death in *N. benthamiana*. Further experiments showed that RsIA_NP8 was upregulated during the early stages of *R. solani* AG1 IA infection, similar to most other effectors, such as smut_2965 and smut_5844 in *Tilletia horrida*, which cause rice kernel smut (Wang et al., 2019). In addition, RsIA_NP8-triggered cell death in *N. benthamiana* depended on its SP. It is interesting that the effector AGLIP1 in *R. solani* AG1 IA also requires its predicted SP to induce cell death (Li et al., 2019). This phenomenon has been observed in *Ustilaginoidea virens* and *T. horrida* (Fang et al., 2016; Wang et al., 2019). In *U. virens*, eight secreted proteins all required SPs to induce cell death in *N. benthamiana* and rice protoplasts (Fang et al., 2016). In *T. horrida*, the full-length effector uan2 was able to trigger cell death in *N. benthamiana*; however, uan2 that lacked SPs was not (Wang et al., 2019). These results show that these proteins have characteristics in common and might function in the extracellular space (Morgan and Kamoun, 2007). A lack of SPs means that these proteins cannot be secreted into the extracellular matrix, where proteins are recognized by plasma membrane receptors (Nie et al., 2019). Another possibility is that these secreted proteins might be translocated into cells after their secretion and recognized by cytoplasmic receptors.

The N-linked glycosylation of secreted proteins is associated with protein folding, stability, quality control, sorting, and secretion and is important for effectors (Helenius and Aebi, 2001, 2004). Most effector glycoproteins are dependent on N-glycosylation for more efficient secretion (Chen et al., 2014). In *U. virens*, mutations on the N-glycosylation sites of effectors UV_1423 and UV_6205 affected the ability to induce cell death. For UV_1423, the mutation of the predicted N-glycosylation site N49G partially prevented the induction of cell death. N39G and N53G mutations of UV_6205 had stronger effects on the inhibition of LUC activity in rice protoplasts (Fang et al., 2016). It is interesting that RsIA_NP8 was predicted by the NetNGlyc 1.0 Server to be N-glycosylated, and three N-glycosylation sites (Asn-92, Asn-129, and Asn-271) were obtained; however, mutations of these predicted N-glycosylation sites did not affect the induction of cell death. Therefore, the function of N-glycosylation of RsIA_NP8 in plant–pathogen interactions needs further study.

Effector proteins containing multiple features, some carrying RxLR motifs, are defined as RxLR effectors, and some possess repeat motifs are repeat-containing proteins (Dou and Zhou, 2012). Repeat motifs possess different types; for example, effectors of the bacterial wilt pathogen, *Ralstonia solanacearum*, RipAP, carry ankyrin repeats (Peeters et al., 2013), RipS1–RipS8 possess HEAT/armadillo repeats (White et al., 2009), and RipG1–RipG7 contain leucine-rich repeats (White et al., 2009). Furthermore, these repeat motifs play important role in the biological function of repeat-containing proteins effectors, such as effector localization, mediating various protein–protein interactions, and providing effector stability (Grove et al., 2008). The effector of the leaf spot pathogen of pepper and tomato, *Xanthomonas euvesicatoria*, XopN, possess seven tandem HEAT/armadillo-like repeats. This effector interacts with the positive regulators of host immunity, TFT1, by HEAT/armadillo-like repeats (Taylor et al., 2012). In our study, RsIA_NP8 contains two new repeat motifs, and these two RPT motifs are required for its ability of induce cell death. Therefore, we suspect that these two RPT motifs play important role in the effector function of RsIA_NP8. This needs further study.

We demonstrated the ability of RsIA_NP8 to activate the *N. benthamiana* immune response by confirming the expression of PR genes, the activation of H_2_O_2_, and callose deposition in *N. benthamiana* leaves. RsIA_NP8 induced ERF1 and LOX at different inoculation times compared to the control. These two genes are related to the activity of jasmonic acid and ethylene, which play an important role in response to necrotrophic pathogens (Wang et al., 2000; Lorenzo et al., 2003). The PR genes PR2b and PR4a were also triggered in *N. benthamiana* leaves following transient expression of RsIA_NP8, which shows that these two PR genes might be involved in resistance to *R. solani*. Hydrogen peroxide and callose deposition has an important role in resistance to plant disease (Mehdy, 1994; Wu et al., 1997; Petersen et al., 2011). In our study, RsIA_NP8 triggered the activation of H_2_O_2_ and callose deposition in *N. benthamiana* leaves. Thus, RsIA_NP8 activated the *N. benthamiana* immune system.

In the study, we identified the secreted protein RsIA_NP8 in the necrotrophic fungus *R. solani*, likely an effector, and showed that it induces cell death or defense responses in *N. benthamiana* leaves. However, the precise molecular mechanisms of how RsIA_NP8 participates in rice–*R. solani* interactions remain to be studied further.

## Materials and Methods

### Strains, Plant Materials, and Growth Conditions

A total of 25 *R. solani* AG1 IA strains were isolated from different areas in China and cultured in PDA medium (200 g potato, 20 g sucrose, 15 g agar, and 1,000 mL distilled water) at 28°C. *Agrobacterium tumefaciens* GV3101 was grown on YEP medium (10 g yeast extract, 10 g 1% tryptone, 5 g NaCl, and 1,000 mL distilled water). The yeast strain YTK12 was cultivated in YPDA medium (10 g yeast extract, 20 g peptone, 20 g glucose, 20 g agar, 0.03 g adenine hemisulfate, and 1,000 mL distilled water). Antibiotics and their concentrations were as follows: kanamycin 100 μg/mL^–1^, ampicillin 100 μg/mL^–1^, and rifampin 25 μg/mL^–1^. Rice cultivar 9311 was provided by the Department of Rice Research Institute of Sichuan Agricultural University. *Nicotiana benthamiana* plants were grown at 12-h/12-h night–day photoperiods at 23°C with 60% relative humidity.

### Candidate Effectors

Signal peptides of secreted proteins were predicted with signalP 4.1^1^ (Petersen et al., 2011), and transmembrane helices were predicted with TMHMM Server version 2.0^2^ Krogh et al., 2001). Effectors were predicted on http://effectorp.csiro.au/. The sequences of encoded proteins <310 aa that contained an N-terminal SP, lacked a transmembrane domain, and were predicted to be effectors on http://effectorp.csiro.au/ were considered candidate effectors (Sperschneider et al., 2018).

### RNA Isolation and Plasmid Construction of *R. solani* Putative Effector Genes

The Fungal RNA Kit (Omega, Biel, Switzerland) was used to extract total RNA from *R. solani* AG1 IA. cDNA was obtained with the Transcriptor First Strand cDNA Synthesis Kit (Roche, Basel, Switzerland). Full-length predicted effector protein-encoding genes were amplified with TransStart FastPfu Fly DNA Polymerase (TransGen Biotech, Beijing, China). All restriction enzymes and ClonExpress enzymes (Vazyme Biotech, Nanjing, China) were used following the manufacturer’s instructions. Primers of these genes were designed with CE Design version 1.03 based on our reference genome sequences and included a *Bam*HI site and a *Stu*I site. Primer sequences are listed in Supplementary Table S2. cDNA obtained from predicted effector genes was gel-purified with a gel purification kit (Omega) and cloned into 35S-PMDC32 expression vector.

### *Agrobacterium tumefaciens*–Mediated Transient Expression

35S-PMDC32 expression vector carrying predicted effector genes was transformed into the *A. tumefaciens* strain GV3101. The bacteria were centrifuged at 5,000 × *g* for 5 min and then resuspended in MES buffer (200 μM acetosyringone, 10 mM MgCl_2_, and 10 mM MES [pH 5.6]). The OD600 of the bacterial suspensions was adjusted to 0.5 and incubated 3 h at room temperature in the dark. The *A. tumefaciens* strain carrying the construct vector was infiltrated into the leaves of *N. benthamiana* at the four-leaf stage. Empty 35S-PMDC32-GFP vector was used as a negative control, and 35S-PMDC32 vector carrying the Bax gene was used as a positive control. A total of 20 leaves from different plants were infiltrated for each control. We observed cell death 4 days after infiltration.

### Function Validation of the SP

We used yeast secretion assay to identify the function of the predicted SP of RsIA_NP8. The SP sequence of RsIA_NP8 was amplified with specific primers by PCR (primer sequences are provided in Supplementary Table S2) and cloned into pSUC2 vectors. The pSUC2 vector carrying the SP sequence of RsIA_NP8 was transformed into the yeast strain YTK12 with the Frozen-EZ Yeast Transformation II Kit (Zymo Research, Irvine, CA, United States). Then, the yeast was grown on CMD-W plates (6.7 g yeast N base without amino acids, 0.75 g tryptophan dropout supplement, 20 g sucrose, 1 g glucose, 15 g agar, and 1,000 mL distilled water, pH 5.8) and YPRAA plates (10 g yeast extract, 20 g peptone, 20 g raffinose, 2 μg antimycin A, l5 g agar, and distilled water 1,000 mL, pH 5.8).

### Site-Directed Mutagenesis

We used splicing overlap extension PCR for site-directed mutagenesis. Two DNA fragments of RsIA_NP8 were amplified from the cDNA of *R. solani* AG1 IA, and the PCR production was cloned into 35S-PMDC32 vectors. The primer sequences used are listed in Supplementary Table S2.

### Quantitative Real-Time Reverse Transcription–Polymerase Chain Reaction

The Fungal RNA Kit (Omega, Norcross, GA, United States) and Spin Column Plant Total RNA Purification Kit (Sangon Biotech, Shanghai, China) were used to extract RNA from *R. solani* AG1 IA strains and *N. benthamiana* leaves, respectively, according to the recommended protocols. The fungal conserved gene 18S rRNA was used as an internal control for data normalization; for *N. benthamiana* leaves, the EF1α gene was used as an internal reference gene to determine relative expression values. cDNA was obtained with the Transcriptor First Strand cDNA Synthesis Kit (Roche). The Bio-Rad CFX96 Real-Time PCR System (Bio-Rad, Foster City, CA, United States) was used for qRT-PCR according to the manufacturer’s instructions. We used the 2^–ΔΔCt^ algorithm to calculate the relative expression of target genes. Four biological replications were performed, and the results were consistent. The primer sequences used are listed in Supplementary Table S2.

### Protein Extraction and Western Blotting

Samples of *N. benthamiana* leaves were obtained 2 days after infiltration and ground in liquid nitrogen. A single-step plant active protein extraction kit (Sangon Biotech) was used to extract protein from the leaves according to the manufacturer’s instructions. The protein samples were separated with 10% sodium dodecyl sulfate–polyacrylamide gel electrophoresis gels and then transferred to nitrocellulose membranes. The membranes were blocked for 1 h at room temperature with 5% milk in TBS-T buffer (50 mM Tris–HCl, pH 7.5; 150 mM NaCl; 0.05% Tween 20) and incubated with anti-FLAG antibody (1:5,000 dilution). Then the membranes were washed three times in TBS-T buffer. The immunoblots were visualized with an eECL Western blotting kit (CWBio) and photographed on X-ray films.

### Trypan Blue Staining, Oxygen Burst, and Callose Deposition Detection

We observed cell death in *N. benthamiana* leaves using trypan blue staining. *Nicotiana benthamiana* leaves were collected 4 days after infiltration and soaked with aldehyde fixative for 24 h. Boiling trypan blue solution (10% lactic acid, 10% glycerol, 10% ddH_2_O, 10% phenol, and 0.67 g trypan blue) was used to dye leaves for 24 h, and then leaves were decolorized for 3 days with 2.5 g/mL chloral hydrate solution (Fernández-Bautista et al., 2016). We observed H_2_O_2_ activation by staining *N. benthamiana* leaves with 3,3′-diaminobenzidine as described previously (Hans et al., 1997). Callose deposition was monitored at 24 h after agroinfiltration for *N. benthamiana*. All experiments were repeated five times.

### VIGS Assay in *N. benthamiana*

*Agrobacterium tumefaciens* GV3101 strains carrying pTRV1 and pTRV2 gene were grown in YEP media (containing appropriate antibiotics) for 36 h, centrifuged, and then suspended in infiltration medium [10 mM MgCl_2_, 10 mM MES (pH 5.7), and 200 μM acetosyringone] mixed at a 1:1 ratio with OD600 = 0.5 for each strain. We then used syringes to infiltrate the cocultures in abaxial side leaves of *N. benthamiana* at the four-leaf stage. We verified the silencing of NbHSP90, NbSERK3, NbRAR1, and NbSGT1 after 20 days of infiltration using qRT-PCR. Three biological replications were performed, and the results were consistent. The primer sequences used are listed in Supplementary Table S2.

### Subcellular Localization

The full-length sequence of RsIA_NP8 was cloned into PHB-YFP vector and then transformed into *A. tumefaciens* GV3101. The transformed *A. tumefaciens* GV3101 infiltrated *N. benthamiana* leaves as described previously. Subcellular localization was detected with laser confocal fluorescence microscopy 2 days after infiltration.

## Data Availability Statement

All datasets generated for this study are included in the article/Supplementary Material.

## Author Contributions

AZ and MW designed the project. MW, YL, and LM performed the experiments. AW and MW wrote the manuscript. XN provided useful advice. All authors read and approved the final manuscript.

## Conflict of Interest

The authors declare that the research was conducted in the absence of any commercial or financial relationships that could be construed as a potential conflict of interest.

## Footnotes

1 http://www.cbs.dtu.dk/services/SignalP/

2 http://www.cbs.dtu.dk/services/TMHMM/

## Supplementary Material

FIGURE S1 Homology of RsIA_NP8 and other fungi.

FIGURE S2 Sequence alignment of RsIA_NP8 in 25 *R. solani* AG1 IA strains.

TABLE S1 The sequence of mutated genes.

TABLE S2 Primer sequences used in this study.
