# Supplementary material for: Identification of the Novel Effector RsIA_NP8 in Rhizoctonia solani AG1 IA That Induces Cell Death and Triggers Defense Responses in Non-Host Plants
Source: Front Microbiol. 2020 Jun 12;11:1115. doi: 10.3389/fmicb.2020.01115 (PMC7303267; doi:10.3389/fmicb.2020.01115)
Supplement: FIGURE S1 — Homology of RsIA_NP8 and other fungi. [file Data_Sheet_1.PDF]

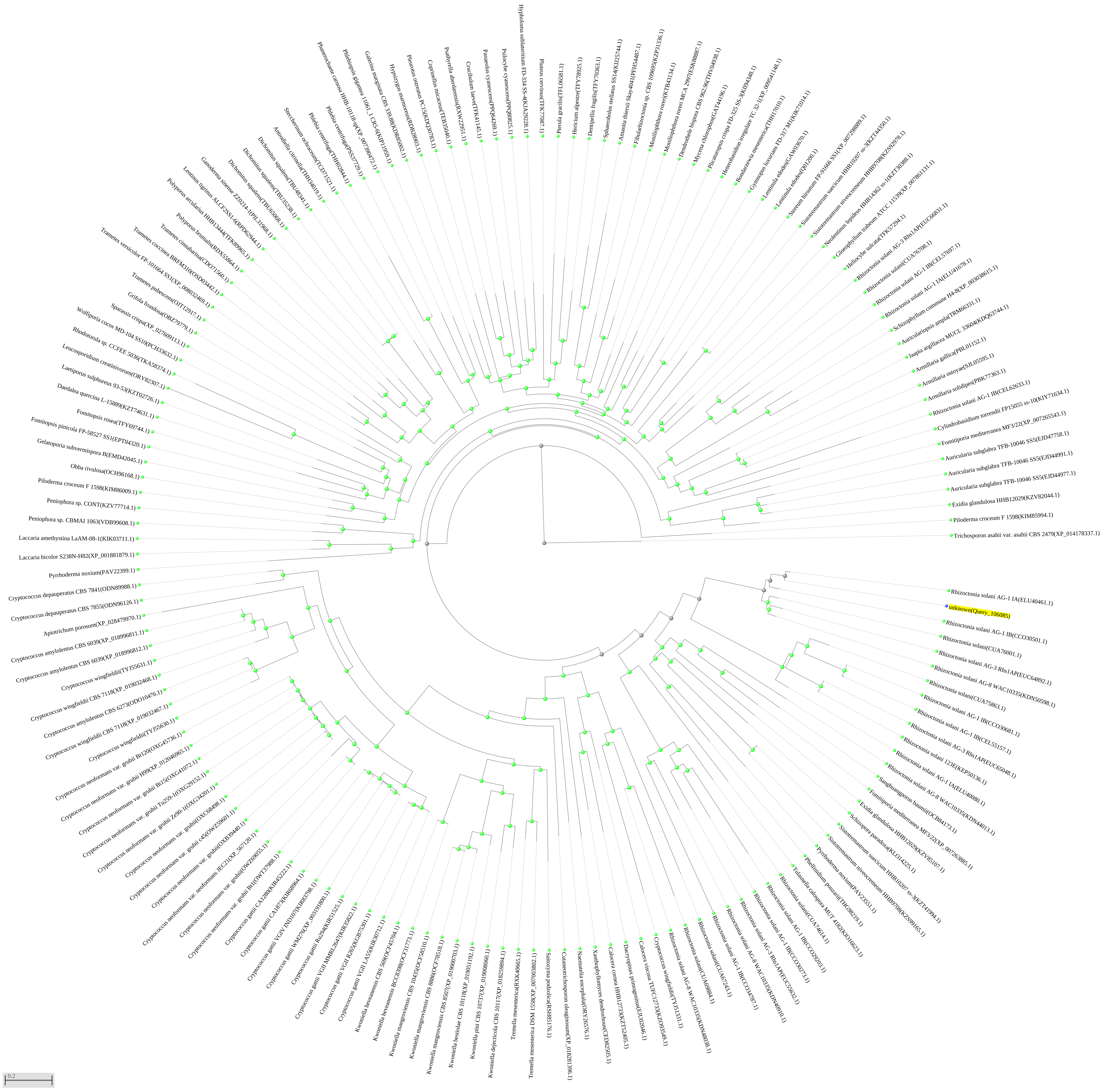

Trichosporon asahii var. asahii CBS 2479(XP_014178337.1)
Piloderma croceum F 1598(KIM85994.1)
Exidia glandulosa HHB12029(KZV82044.1)
Auricularia subglabra TFB-10046 SS5(EJD44977.1)
Auricularia subglabra TFB-10046 SS5(EJD44991.1)
Auricularia subglabra TFB-10046 SS5(EJD47758.1)
Fomitiporia mediterranea MF3/22(XP_007265543.1)
Cylindrobasidium torrendii FP15055 ss-10(KIY71634.1)
Rhizoctonia solani AG-1 IB(CEL62633.1)
Armillaria solidipes(PBK77363.1)
Armillaria ostoyae(SJL05595.1)
Armillaria gallica(PBL01152.1)
Jaapia argillacea MUCL 33604(KDQ63744.1)
Auriculariopsis ampla(TRM66331.1)
Schizophyllum commune H4-8(XP_003038615.1)
Rhizoctonia solani AG-1 IA(ELU41678.1)
Rhizoctonia solani AG-1 IB(CEL57697.1)
Rhizoctonia solani(CUA76708.1)
Rhizoctonia solani AG-3 Rhs1AP(EUC66831.1)
Heliocybe sulcata(TFK57294.1)
Gloeophyllum trabeum ATCC 11539(XP_007861131.1)
Neolentinus lepideus HHB14362 ss-1(KZT30288.1)
Sistotremastrum niveocremeum HHB9708(KZS92978.1)
Sistotremastrum suecicum HHB10207 ss-3(KZT44350.1)
Stereum hirsutum FP-91666 SS1(XP_007298889.1)
Lentinula edodes(QI01280.1)
Lentinula edodes(GAW03670.1)
Gymnopus luxurians FD-317 M1(KIK71014.1)
Bondarzewia mesenterica(THH17010.1)
Heterobasidion irregulare TC 32-1(XP_009541148.1)
Plicaturopsis crispa FD-325 SS-3(KII94348.1)
Mycena chlorophos(GAT44196.1)
Dendrothele bispora CBS 962.96(THV04938.1)
Moniliophthora roreri MCA 2997(ESK88887.1)
Moniliophthora roreri(KTB43134.1)
Fibularhizoctonia sp. CBS 109695(KZP31336.1)
Amanita thiersii Skay4041(PFH54487.1)
Sphaerobolus stellatus SS14(KIJ25744.1)
Dentipellis fragilis(TFY70363.1)
Hericium alpestre(TFY78925.1)
Pterula gracilis(TFL06581.1)
Pluteus cervinus(TFK77087.1)
Hypholoma sublateritium FD-334 SS-4(KJA29228.1)
Psilocybe cyanescens(PPQ80825.1)
Panaeolus cyanescens(PPQ64269.1)
Crucibulum laeve(TFK41145.1)
Psathyrella aberdarensis(RXW22951.1)
Coprinellus micaceus(TEB35040.1)
Pleurotus ostreatus PC15(KDQ30783.1)
Hypsizygus marmoreus(RDB28903.1)
Galerina marginata CBS 339.88(KDR65007.1)
Phlebiopsis gigantea 11061_1 CR5-6(KIP11959.1)
Phanerochaete carnosa HHB-10118-sp(XP_007390472.1)
Phlebia centrifuga(PSS37729.1)
Phlebia centrifuga(THH02844.1)
Steccherinum ochraceum(TCD71521.1)
Antrodiella citrinella(THH34019.1)
Dichomitus squalens(TBU48341.1)
Dichomitus squalens(TBU35238.1)
Dichomitus squalens(TBU65068.1)
Ganoderma sinense ZZ0214-1(PIL31968.1)
Lentinus tigrinus ALCF2SS1-6(RPD62944.1)
Polyporus arcularius HHB13444(TFK89965.1)
Polyporus brumalis(RDX55864.1)
Trametes cinnabarina(CDO71560.1)
Trametes coccinea BRFM310(OSD03442.1)
Trametes versicolor FP-101664 SS1(XP_008032469.1)
Trametes pubescens(OJT12917.1)
Grifola frondosa(OBZ79779.1)
Sparassis crispa(XP_027609113.1)
Wolfiporia cocos MD-104 SS10(PCH33632.1)
Rhodotorula sp. CCFEE 5036(TKA58374.1)
Leucosporidium creatinivorum(ORY82307.1)
Laetiporus sulphureus 93-53(KZT02726.1)
Daedalea quercina L-15889(KZT74631.1)
Fomitopsis rosea(TFY69744.1)
Fomitopsis pinicola FP-58527 SS1(EPT04320.1)
Gelatoporia subvermispora B(EMD42045.1)
Obba rivulosa(OCH96168.1)
Piloderma croceum F 1598(KIM86009.1)
Peniophora sp. CONT(KZV77714.1)
Peniophora sp. CBMAI 1063(VDB99608.1)
Laccaria amethystina LaAM-08-1(KIK03711.1)
Laccaria bicolor S238N-H82(XP_001881879.1)
Pyrrhoderma noxium(PAV22399.1)
Cryptococcus depauperatus CBS 7841(ODN89988.1)
Cryptococcus depauperatus CBS 7855(ODN96126.1)
Apiotrichum porosum(XP_028479970.1)
Cryptococcus amylolentus CBS 6039(XP_018996811.1)
Cryptococcus amylolentus CBS 6039(XP_018996812.1)
Cryptococcus wingfieldii(TYJ55631.1)
Cryptococcus wingfieldii CBS 7118(XP_019032468.1)
Cryptococcus amylolentus CBS 6273(ODO10476.1)
Cryptococcus wingfieldii CBS 7118(XP_019032467.1)
Cryptococcus wingfieldii(TYJ55630.1)
Cryptococcus neoformans var. grubii Bt120(OXG45736.1)
Cryptococcus neoformans var. grubii H99(XP_012046965.1)
Cryptococcus neoformans var. grubii Bt15(OXG41072.1)
Cryptococcus neoformans var. grubii Tu259-1(OXG29152.1)
Cryptococcus neoformans var. grubii Ze90-1(OXC68498.1)
Cryptococcus neoformans var. grubii c45(OWZ59601.1)
Cryptococcus neoformans var. grubii(OXB39440.1)
Cryptococcus neoformans var. neoformans JEC21(XP_567120.1)
Cryptococcus neoformans var. grubii(OWZ69055.1)
Cryptococcus neoformans var. grubii Bt1(OWT37988.1)
Cryptococcus gattii CA1280(KIR45222.1)
Cryptococcus gattii CA1873(KIR68964.1)
Cryptococcus gattii VGIV IND107(KIR83798.1)
Cryptococcus gattii WM276(XP_003191800.1)
Cryptococcus gattii Ru294(KIR51525.1)
Cryptococcus gattii VGII MMRL2647(KIR35822.1)
Cryptococcus gattii VGII R265(KGB75301.1)
Cryptococcus gattii VGII LA55(KIR30712.1)
Kwoniella heveanensis CBS 569(OCF45704.1)
Kwoniella heveanensis BCC8398(OCF31773.1)
Kwoniella mangroviensis CBS 10435(OCF58510.1)
Kwoniella mangroviensis CBS 8886(OCF78518.1)
Kwoniella mangroviensis CBS 8507(XP_019000703.1)
Kwoniella bestiolae CBS 10118(XP_019051192.1)
Kwoniella pini CBS 10737(XP_019008060.1)
Kwoniella dejecticola CBS 10117(XP_018259894.1)
Tremella mesenterica(RXK40665.1)
Tremella mesenterica DSM 1558(XP_007003802.1)
Saitozyma podzolica(RSH85176.1)
Cutaneotrichosporon oleaginosum(XP_018281396.1)
Naematelia encephala(ORY26576.1)
Xanthophyllomyces dendrorhous(CED82505.1)
Calocera cornea HHB12733(KZT52405.1)
Dacryopinax primogenitus(EJU03046.1)
Calocera viscosa TUFC12733(KZO93549.1)
Cryptococcus wingfieldii(TYJ51311.1)
Rhizoctonia solani AG-8 WAC10335(KDN48038.1)
Rhizoctonia solani(CUA69884.1)
Rhizoctonia solani(CUA67243.1)
Rhizoctonia solani AG-1 IB(CCO34787.1)
Rhizoctonia solani AG-8 WAC10335(KDN40910.1)
Rhizoctonia solani AG-3 Rhs1AP(EUC55652.1)
Rhizoctonia solani AG-1 IB(CCO30273.1)
Rhizoctonia solani(CUA74614.1)
Tulasnella calospora MUT 4182(KIO29203.1)
Phellinidium pouzarii(THG98219.1)
Pyrrhoderma noxium(PAV23551.1)
Sistotremastrum niveocremeum HHB9708(KZS99165.1)
Sistotremastrum suecicum HHB10207 ss-3(KZT41994.1)
Schizopora paradoxa(KLO14225.1)
Exidia glandulosa HHB12029(KZV85107.1)
Fomitiporia mediterranea MF3/22(XP_007263885.1)
Sanghuangporus baumii(OCB84173.1)
Rhizoctonia solani AG-8 WAC10335(KDN44013.1)
Rhizoctonia solani AG-1 IA(ELU40080.1)
Rhizoctonia solani 123E(KEP50136.1)
Rhizoctonia solani AG-3 Rhs1AP(EUC65048.1)
Rhizoctonia solani AG-1 IB(CEL55157.1)
Rhizoctonia solani AG-1 IB(CCO30681.1)
Rhizoctonia solani(CUA75863.1)
Rhizoctonia solani AG-8 WAC10335(KDN50598.1)
Rhizoctonia solani AG-3 Rhs1AP(EUC64892.1)
Rhizoctonia solani(CUA76001.1)
Rhizoctonia solani AG-1 IB(CCO30501.1)
unknown(Query_106085)
Rhizoctonia solani AG-1 IA(ELU40461.1)
0.2
