# Supplementary material for: Identification of the Novel Effector RsIA_NP8 in Rhizoctonia solani AG1 IA That Induces Cell Death and Triggers Defense Responses in Non-Host Plants
Source: Front Microbiol. 2020 Jun 12;11:1115. doi: 10.3389/fmicb.2020.01115 (PMC7303267; doi:10.3389/fmicb.2020.01115)
Supplement: FIGURE S2 — Sequence alignment of RsIA_NP8 in 25 R. solani AG1 IA strains. [file Data_Sheet_2.PDF]

Section 1

	(1)	1 10 20 30 40 50 60 70 87
AG1IA	(1)	ATGCGTTTTGCGACCGTAGCCTCTGCTGTGACCGTCTTGGTCASTGCTGCTGCTCCCGCCGTGGCCACCTTCAATCTTCGCTGCAAT
CC7-1	(1)	ATGCGTTTTGCGACCGTAGCCTCTGCTGTGACCGTCTTGGTCAGTGCTGCTGCTCCCGCCGTGGCCACCTTCAATCTTCGCTGCAAT
NJ1-1	(1)	ATGCGTTTTGCGACCGTAGCCTCTGCTGTGACCGTCTTGGTCAGTGCTGCTGCTCCCGCCGTGGCCACCTTCAATCTTCGCTGCAAT
NJ2-1	(1)	ATGCGTTTTGCGACCGTAGCCTCTGCTGTGACCGTCTTGGTCAGTGCTGCTGCTCCCGCCGTGGCCACCTTCAATCTTCGCTGCAAT
ZZYY3-1-2	(1)	ATGCGTTTTGCGACCGTAGCCTCTGCTGTGACCGTCTTGGTCAGTGCTGCTGCTCCCGCCGTGGCCACCTTCAATCTTCGCTGCAAT
CC2-1	(1)	ATGCGTTTTGCGACCGTAGCCTCTGCTGTGACCGTCTTGGTCAGTGCTGCTGCTCCCGCCGTGGCCACCTTCAATCTTCGCTGCAAT
CQ3-2	(1)	ATGCGTTTTGCGACCGTAGCCTCTGCTGTGACCGTCTTGGTCAGTGCTGCTGCTCCCRCCGTGGCCACCTTCAATCTTCGCTGCAAT
FJ6	(1)	ATGCGTTTTGCGACCGTAGCCTCTGCTGTGACCGTCTTGGTCAGTGCTGCTGCTCCCGCCGTGGCCACCTTCAATCTTCGCTGCAAT
FJ7-1	(1)	ATGCGTTTTGCGACCGTAGCCTCTGCTGTGACCGTCTTGGTCAGTGCTGCTGCTCCCGCCGTGGCCACCTTCAATCTTCGCTGCAAT
GD3-1	(1)	ATGCGTTTTGCGACCGTAGCCTCTGCTGTGACCGTCTTGGTCAGTGCTGCTGCTCCCGCCGTGGCCACCTTCAATCTTCGCTGCAAT
GZ4-1	(1)	ATGCGTTTTGCGACCGTAGCCTCTGCTGTGACCGTCTTGGTCAGTGCTGCTGCTCCCGCCGTGGCCACCTTCAATCTTCGCTGCAAT
GZ8-1	(1)	ATGCGTTTTGCGACCGTAGCCTCTGCTGTGACCGTCTTGGTCAGTGCTGCTGCTCCCGCCGTGGCCACCTTCAATCTTCGCTGCAAT
HB1-1	(1)	ATGCGTTTTGCGACCGTAGCCTCTGCTGTGACCGTCTTGGTCAGTGCTGCTGCTCCCGCCGTGGCCACCTTCAATCTTCGCTGCAAT
MA-1-1-1	(1)	ATGCGTTTTGCGACCGTAGCCTCTGCTGTGACCGTCTTGGTCAGTGCTGCTGCTCCCGCCGTGGCCACCTTCAATCTTCGCTGCAAT
MB-2-1-1	(1)	ATGCGTTTTGCGACCGTRGCCTCTGCTGTGACCGTCTTGGTCAGTGCTGCTGCTCCCGCCGTGGCCACCTTCAATCTTCGCTGCAAT
HN2-2	(1)	ATGCGTTTTGCGACCGTAGCCTCTGCTGTGACCGTCTTGGTCAGTGCTGCTGCTCCCGCCGTGGCCACCTTCAATCTTCGCTGCAAT
WJ1-1	(1)	ATGCGTTTTGCGACCGTAGCCTCTGCTGTGACCGTCTTGGTCAGTGCTGCTGCTCCCGCCGTGGCCACCTTCAATCTTCGCTGCAAT
ZZYY2-1-1	(1)	ATGCGTTTTGCGACCGTAGCCTCTGCTGTGACCGTCTTGGTCAGTGCTGCTGCTCCCGCCGTGGCCACCTTCAATCTTCGCTGCAAT
HZFY4-1-1	(1)	ATGCGTTTTGCGACCGTAGCCTCTGCTGTGACCGTCTTGGTCAGTGCTGCTGCTCCCACCGTGGCCACCTTCAATCTTCGCTGCAAT
AG1IA-05500	(1)	ATGCGTTTTGCGACCGTAGCCTCTGCTGTGACCGTCTTGGTCAGTGCTGCTGCTCCCGCCGTGGCCACCTTCAATCTTCGCTGCAAT
AH3	(1)	ATGCGTTTTGCGACCGTAGCCTCTGCTGTGACCGTCTTGGTCAGTGCTGCTGCTCCCGCCGTGGCCACCTTCAATCTTCGCTGCAAT
HZFY5-2-1	(1)	ATGCGTTTTGCGACCGTAGCCTCTGCTGTGACCGTCTTGGTCAGTGCTGCTGCTCCCGCCGTGGCCACCTTCAATCTTCGCTGCAAT
FM1-1	(1)	ATGCGTTTTGCGACCGTAGCCTCTGCTGTGACCGTCTTGGTCAGTGCTGCTGCTCCCGCCGTGGCCACCTTCAATCTTCGCTGCAAT
FM2-1	(1)	ATGCGTTTTGCGACCGTAGCCTCTGCTGTGACCGTCTTGGTCAGTGCTGCTGCTCCCGCCGTGGCCACCTTCAATCTTCGCTGCAAT
SY1-1	(1)	ATGCGTTTTGCGACCGTAGCCTCTGCTGTGACCGTCTTGGTCAGTGCTGCTGCTCCCGCCGTGGCCACCTTCAATCTTCGCTGCAAT
SY5-1	(1)	ATGCGTTTTGCGACCGTAGCCTCTGCTGTGACCGTCTTGGTCAGTGCTGCTGCTCCCGCCGTGGCCACCTTCAATCTTCGCTGCAAT
WC2-1	(1)	ATGCGTTTTGCGACCGTAGCCTCTGCTGTGACCGTCTTGGTCAGTGCTGCTGCTCCCGCCGTGGCCACCTTCAATCTTCGCTGCAAT
Consensus	(1)	ATGCGTTTTGCGACCGTAGCCTCTGCTGTGACCGTCTTGGTCAGTGCTGCTGCTCCCGCCGTGGCCACCTTCAATCTTCGCTGCAAT

Section 2

	(88)	88 100 110 120 130 140 150 160 174
AG1IA	(88)	CTCGGAAGCTGGTACTGGAAGGACAAGGACTGCTGCATTAAGGTTGGCGGACCCATATCTCCGCCCAGCCCTCCCTCTGGGAATGCT
CC7-1	(88)	CTCGGAAGCTGGTACTGGAAGGACAAGGACTGCTGCATTAAGGTTGGCGGACCCATATCTCCGCCCAGCCCTCCCTCTGGGAATGCT
NJ1-1	(88)	CTCGGAAGCTGGTACTGGAAGGACAAGGACTGCTGCATTAAGGTTGGCGGACCCATATCTCCGCCCAGCCCTCCCTCTGGGAATGCT
NJ2-1	(88)	CTCGGAAGCTGGTACTGGAAGGACAAGGACTGCTGCATTAAGGTTGGCGGACCCATATCTCCGCCCAGCCCTCCCTCTGGGAATGCT
ZZYY3-1-2	(88)	CTCGGAAGCTGGTACTGGAAGGACAAGGACTGCTGCATTAAGGTTGGCGGACCCATATCTCCGCCCAGCCCTCCCTCTGGGAATGCT
CC2-1	(88)	CTCGGAAGCTGGTACTGGAAGGACAAGGACTGCTGCATTAAGGTTGGCGGACCCATATCTCCGCCCAGCCCTCCCTCTGGGAATGCT
CQ3-2	(88)	CTCGGAAGCTGGTACTGGAAGGACAAGGACTGCTGCATTAAGGTTGGCGGACCCATATCTCCGCCCAGCCCTCCCTCTGGGAATGCT
FJ6	(88)	CTCGGAAGCTGGTACTGGAAGGACAAGGACTGCTGCATTAAGGTTGGCGGACCCATATCTCCGCCCAGCCCTCCCTCTGGGAATGCT
FJ7-1	(88)	CTCGGAAGCTGGTACTGGAAGGACAAGGACTGCTGCATTAAGGTTGGCGGACCCATATCTCCGCCCAGCCCTCCCTCTGGGAATGCT
GD3-1	(88)	CTCGGAAGCTGGTACTGGAAGGACAAGGACTGCTGCATTAAGGTTGGCGGACCCATATCTCCGCCCAGCCCTCCCTCTGGGAATGCT
GZ4-1	(88)	CTCGGAAGCTGGTACTGGAAGGACAAGGACTGCTGCATTAAGGTTGGCGGACCCATATCTCCGCCCAGCCCTCCCTCTGGGAATGCT
GZ8-1	(88)	CTCGGAAGCTGGTACTGGAAGGACAAGGACTGCTGCATTAAGGTTGGCGGACCCATATCTCCGCCCAGCCCTCCCTCTGGGAATGCT
HB1-1	(88)	CTCGGAAGCTGGTACTGGAAGGACAAGGACTGCTGCATTAAGGTTGGCGGACCCATATCTCCGCCCAGCCCTCCCTCTGGGAATGCT
MA-1-1-1	(88)	CTCGGAAGCTGGTACTGGAAGGACAAGGACTGCTGCATTAAGGTTGGCGGACCCATATCTCCGCCCAGCCCTCCCTCTGGGAATGCT
MB-2-1-1	(88)	CTCGGAAGCTGGTACTGGAAGGACAAGGACTGCTGCATTAAGGTTGGCGGACCCATATCTCCGCCCAGCCCTCCCTCTGGGAATGCT
HN2-2	(88)	CTCGGAAGCTGGTACTGGAAGGACAAGGACTGCTGCATTAAGGTTGGCGGACCCATATCTCCGCCCAGCCCTCCCTCTGGGAATGCT
WJ1-1	(88)	CTCGGAAGCTGGTACTGGAAGGACAAGGACTGCTGCATTAAGGTTGGCGGACCCATATCTCCGCCCAGCCCTCCCTCTGGGAATGCT
ZZYY2-1-1	(88)	CTCGGAAGCTGGTACTGGAAGGACAAGGACTGCTGCATTAAGGTTGGCGGACCCATATCTCCGCCCAGCCCTCCCTCTGGGAATGCT
HZFY4-1-1	(88)	CTCGGAAGCTGGTACTGGAAGGACAAGGACTGCTGCATTAAGGTTGGCGGACCCATATCTCCGCCCAGCCCTCCCTCTGGGAATGCT
AG1IA-05500	(88)	CTCGGAAGCTGGTACTGGAAGGACAAGGACTGCTGCATTAAGGTTGGCGGACCCATATCTCCGCCCAGCCCTCCCTCTGGGAATGCT
AH3	(88)	CTCGGAAGCTGGTACTGGAAGGACAAGGACTGCTGCATTAAGGTTGGCGGACCCATATCTCCGCCCAGCCCTCCCTCTGGGAATGCT
HZFY5-2-1	(88)	CTCGGAAGCTGGTACTGGAAGGACAAGGACTGCTGCATTAAGGTTGGCGGACCCATATCTCCGCCCAGCCCTCCCTCTGGGAATGCT
FM1-1	(88)	CTCGGAAGCTGGTACTGGAAGGACAAGGACTGCTGCATTAAGGTTGGCGGACCCATATCTCCGCCCAGCCCTCCCTCTGGGAATGCT
FM2-1	(88)	CTCGGAAGCTGGTACTGGAAGGACAAGGACTGCTGCATTAAGGTTGGCGGACCCATATCTCCGCCCAGCCCTCCCTCTGGGAATGCT
SY1-1	(88)	CTCGGAAGCTGGTACTGGAAGGACAAGGACTGCTGCATTAAGGTTGGCGGACCCATATCTCCGCCCAGCCCTCCCTCTGGGAATGCT
SY5-1	(88)	CTCGGAAGCTGGTACTGGAAGGACAAGGACTGCTGCATTAAGGTTGGCGGACCCATATCTCCGCCCAGCCCTCCCTCTGGGAATGCT
WC2-1	(88)	CTCGGAAGCTGGTACTGGAAGGACAAGGACTGCTGCATTAAGGTTGGCGGACCCATATCTCCGCCCAGCCCTCCCTCTGGGAATGCT
Consensus	(88)	CTCGGAAGCTGGTACTGGAAGGACAAGGACTGCTGCATTAAGGTTGGCGGACCCATATCTCCGCCCAGCCCTCCCTCTGGGAATGCT

Section 3

(175)	175 180 190 200 210 220 230 240 250 261
AG1IA (175)	TGCCCCAACTTGTGGTTCTGGAACAAGGACACCAACTGCTGTGCCCCTCGGTACACTCCCCCTTACAACCAGCCATCGTGCAGCAAC
CC7-1 (175)	TGCCCCAACTTGTGGTTCTGGAACAAGGACACCAACTGCTGTGCCCCTCGGTACACTCCCCCTTACAACCAGCCATCGTGCAGCAAC
NJ1-1 (175)	TGCCCCAACTTGTGGTTCTGGAACAAGGACACCAACTGCTGTGCCCCTCGGTACACTCCCCCTTACAACCAGCCATCGTGCAGCAAC
NJ2-1 (175)	TGCCCCAACTTGTGGTTCTGGAACAAGGACACCAACTGCTGTGCCCCTCGGTACACTCCCCCTTACAACCAGCCATCGTGCAGCAAC
ZZYY3-1-2 (175)	TGCCCCAACTTGTGGTTCTGGAACAAGGACACCAACTGCTGTGCCCCTCGGTACACTCCCCCTTACAACCAGCCATCGTGCAGCAAC
CC2-1 (175)	TGCCCCAACTTGTGGTTCTGGAACAAGGACACCAACTGCTGTGCCCCTCGGTACACTCCCCCTTACAACCAGCCATCGTGCAGCAAC
CQ3-2 (175)	TGCCCCAACTTGTGGTTCTGGAACAAGGACACCAACTGCTGTGCCCCTCGGTACACTCCCCCTTACAACCAGCCATCGTGCAGCAAC
FJ6 (175)	TGCCCCAACTTGTGGTTCTGGAACAAGGACACCAACTGCTGTGCCCCTCGGTACACTCCCCCTTACAACCAGCCATCGTGCAGCAAC
FJ7-1 (175)	TGCCCCAACTTGTGGTTCTGGAACAAGGACACCAACTGCTGTGCCCCTCGGTACACTCCCCCTTACAACCAGCCATCGTGCAGCAAC
GD3-1 (175)	TGCCCCAACTTGTGGTTCTGGAACAAGGACACCAACTGCTGTGCCCCTCGGTACACTCCCCCTTACAACCAGCCATCGTGCAGCAAC
GZ4-1 (175)	TGCCCCAACTTGTGGTTCTGGAACAAGGACACCAACTGCTGTGCCCCTCGGTACACTCCCCCTTACAACCAGCCATCGTGCAGCAAC
GZ8-1 (175)	TGCCCCAACTTGTGGTTCTGGAACAAGGACACCAACTGCTGTGCCCCTCGGTACACTCCCCCTTACAACCAGCCATCGTGCAGCAAC
HB1-1 (175)	TGCCCCAACTTGTGGTTCTGGAACAAGGACACCAACTGCTGTGCCCCTCGGTACACTCCCCCTTACAACCAGCCATCGTGCAGCAAC
MA-1-1-1 (175)	TGCCCCAACTTGTGGTTCTGGAACAAGGACACCAACTGCTGTGCCCCWCGGTACACTCCCCCTTACAACCAGCCATCGTGCAGCAAC
MB-2-1-1 (175)	TGCCCCAACTTGTGGTTCTGGAACAAGGACACCAACTGCTGTGCCCCTCGGTACACTCCCCCTTACAACCAGCCATCGTGCAGCAAC
HN2-2 (175)	TGCCCCAACTTGTGGTTCTGGAACAAGGACACCAACTGCTGTGCCCCTCGGTACACTCCCCCTTACAACCAGCCATCGTGCAGCAAC
WJ1-1 (175)	TGCCCCAACTTGTGGTTCTGGAACAAGGACACCAACTGCTGTGCCCCTCGGTACACTCCCCCTTACAACCAGCCATCGTGCAGCAAC
ZZYY2-1-1 (175)	TGCCCCAACTTGTGGTTCTGGAACAAGGACACCAACTGCTGTGCCCCTCGGTACACTCCCCCTTACAACCAGCCATCGTGCAGCAAC
HZFY4-1-1 (175)	TGCCCCAACTTGTGGTTCTGGAACAAGGACACCAACTGCTGTGCCCCTCGGTACACTCCCCCTTACAACCAGCCATCGTGCAGCAAC
AG1IA-05500 (175)	TGCCCCAACTTGTGGTTCTGGAACAAGGACACCAACTGCTGTGCCCCTCGGTACACTCCCCCTTACAACCAGCCATCGTGCAGCAAC
AH3 (175)	TGCCCCAACTTGTGGTTCTGGAACAAGGACACCAACTGCTGTGCCCCTCGGTACACTCCCCCTTACAACCAGCCATCGTGCAGCAAC
HZFY5-2-1 (175)	TGCCCCAACTTGTGGTTCTGGAACAAGGACACCAACTGCTGTGCCCCTCGGTACACTCCCCCTTACAACCAGCCATCGTGCAGCAAC
FM1-1 (175)	TGCCCCAACTTGTGGTTCTGGAACAAGGACACCAACTGCTGTGCCCCTCGGTACACTCCCCCTTACAACCAGCCATCGTGCAGCAAC
FM2-1 (175)	TGCCCCAACTTGTGGTTCTGGAACAAGGACACCAACTGCTGTGCCCCTCGGTACACTCCCCCTTACAACCAGCCATCGTGCAGCAAC
SY1-1 (175)	TGCCCCAACTTGTGGTTCTGGAACAAGGACACCAACTGCTGTGCCCCTCGGTACACTCCCCCTTACAACCAGCCATCGTGCAGCAAC
SY5-1 (175)	TGCCCCAACTTGTGGTTCTGGAACAAGGACACCAACTGCTGTGCCCCTCGGTACACTCCCCCTTACAACCAGCCATCGTGCAGCAAC
WC2-1 (175)	TGCCCCAACTTGTGGTTCTGGAACAAGGACACCAACTGCTGTGCCCCTCGGTACACTCCCCCTTACAACCAGCCATCGTGCAGCAAC
Consensus (175)	TGCCCCAACTTGTGGTTCTGGAACAAGGACACCAACTGCTGTGCCCCTCGGTACACTCCCCCTTACAACCAGCCATCGTGCAGCAAC

Section 4

```
                 262     270       280       290       300       310       320       330             348
            (262) GGATGGGTCTGGAATCCCAGCACATACTCTTGTCGCCCGCCTGCTACACCCCCACCAAAGAACAAYGATTGTGGCGMCAACAAATGG
AG1IA       (262) GGATGGGTCTGGAATCCCAGCACATACTCTTGTCGCCCGCCTGCTACACCCCCACCAAAGAACAAYGATTGTGGCGMCAACAAATGG
CC7-1       (262) GGATGGGTCTGGAATCCCAGCACATACTCTTGTCGCCCGCCTGCTACACCCCCACCAAAGAACAAYGATTGTGGCGMCAACAAATGG
NJ1-1       (262) GGATGGGTCTGGAATCCCAGCACATACTCTTGTCGCCCGCCTGCTACACCCCCACCAAAGAACAAYGATTGTGGCGMCAACAAATGG
NJ2-1       (262) GGATGGGTCTGGAATCCCAGCACATACTCTTGTCGCCCGCCTGCTACACCCCCACCAAAGAACAAYGATTGTGGCGMCAACAAATGG
ZZYY3-1-2   (262) GGATGGGTCTGGAATCCCAGCACATACTCTTGTCGCCCGCCTGCTACACCCCCACCAAAGAACAAYGATTGTGGCGMCAACAAATGG
CC2-1       (262) GGATGGGTCTGGAATCCCAGCACATACTCTTGTCGCCCGCCTGCTACACCCCCACCAAAGAACAAYGATTGTGGCGMCAACAAATGG
CQ3-2       (262) GGATGGGTCTGGAATCCCAGCACATACTCTTGTCGCCCGCCTGCTACACCCCCACCAAAGAACAATGATTGTGGCGACAACAAATGG
FJ6         (262) GGATGGGTCTGGAATCCCAGCACATACTCTTGTCGCCCGCCTGCTACACCCCCACCAAAGAACAATGATTGTGGCGACAACAAATGG
FJ7-1       (262) GGATGGGTCTGGAATCCCAGCACATACTCTTGTCGCCCGCCTGCTACACCCCCACCAAAGAACAATGATTGTGGCGACAACAAATGG
GD3-1       (262) GGATGGGTCTGGAATCCCAGCACATACTCTTGTCGCCCGCCTGCTACACCCCCACCAAAGAACAACGATTGTGGCGACAACAAATGG
GZ4-1       (262) GGATGGGTCTGGAATCCCAGCACATACTCTTGTCGCCCGCCTGCTACACCCCCACCAAAGAACAACGATTGTGGCGMCAACAAATGG
GZ8-1       (262) GGATGGGTCTGGAATCCCAGCACATACTCTTGTCGCCCGCCTGCTACACCCCCACCAAAGAACAACGATTGTGGCGMCAACAAATGG
HB1-1       (262) GGATGGGTCTGGAATCCCAGCACATACTCTTGTCGCCCGCCTGCTACACCCCCACCAAAGAACAACGATTGTGGCGMCAACAAATGG
MA-1-1-1    (262) GGATGGGTCTGGAATCCCAGCACATACTCTTGTCGCCCGCCTGCTACACCCCCACCAAAGAACAACGATTGTGGCGACAACAAATGG
MB-2-1-1    (262) GGATGGGTCTGGAATCCCAGCACATACTCTTGTCGCCCGCCTGCTACACCCCCACCAAAGAACAACGATTGTGGCGACAACAAATGG
HN2-2       (262) GGATGGGTCTGGAATCCCAGCACATACTCTTGTCGCCCGCCTGCTACACCCCCACCAAAGAACAATGATTGTGGCGACAACAAATGG
WJ1-1       (262) GGATGGGTCTGGAATCCCAGCACATACTCTTGTCGCCCGCCTGCTACACCCCCACCAAAGAACAATGATTGTGGCGACAACAAATGG
ZZYY2-1-1   (262) GGATGGGTCTGGAATCCCAGCACATACTCTTGTCGCCCGCCTGCTACACCCCCACCAAAGAACAATGATTGTGGCGACAACAAATGG
HZFY4-1-1   (262) GGATGGGTCTGGAATCCCAGCACATACTCTTGTCGCCCGCCTGCTACACCCCCACCAAAGAACAATGATTGTGGCGACAACAAATGG
AG1IA-05500 (262) GGATGGGTCTGGAATCCCAGCACATACTCTTGTCGCCCGCCTGCTACACCCCCACCAAAGAACAACGATTGTGGCGCCAACAAATGG
AH3         (262) GGATGGGTCTGGAATCCCAGCACATACTCTTGTCGCCCGCCTGCTACACCCCCACCAAAGAACAACGATTGTGGCGCCAACAAATGG
HZFY5-2-1   (262) GGATGGGTCTGGAATCCCAGCACATACTCTTGTCGCCCGCCTGCTACACCCCCACCAAAGAACAACGATTGTGGCGCCAACAAATGG
FM1-1       (262) GGATGGGTCTGGAATCCCAGCACATACTCTTGTCGCCCGCCTGCTACACCCCCACCAAAGAACAACGATTGTGGCGCCAACAAATGG
FM2-1       (262) GGATGGGTCTGGAATCCCAGCACATACTCTTGTCGCCCGCCTGCTACACCCCCACCAAAGAACAACGATTGTGGCGCCAACAAATGG
SY1-1       (262) GGATGGGTCTGGAATCCCAGCACATACTCTTGTCGCCCGCCTGCTACACCCCCACCAAAGAACAACGATTGTGGCGCCAACAAATGG
SY5-1       (262) GGATGGGTCTGGAATCCCAGCACATACTCTTGTCGCCCGCCTGCTACACCCCCACCAAAGAACAACGATTGTGGCGCCAACAAATGG
WC2-1       (262) GGATGGGTCTGGAATCCCAGCACATACTCTTGTCGCCCGCCTGCTACACCCCCACCAAAGAACAACGATTGTGGCGCCAACAAATGG
Consensus   (262) GGATGGGTCTGGAATCCCAGCACATACTCTTGTCGCCCGCCTGCTACACCCCCACCAAAGAACAACGATTGTGGCG CAACAAATGG
```

Section 5

(349)	349 360 370 380 390 400 410 420 435
AG1IA (349)	TGGTGGGGTGGTCGCTCTTGCTGTCTTCCTCACGGAAACCCATCGCCGCCGCTCCCTCCTCGCGGAAACAACTGCCCACCCAGTTGG
CC7-1 (349)	TGGTGGGGTGGTCGCTCTTGCTGTCTTCCTCACGGAAACCCATCGCCGCCGCTCCCTCCTCGCGGAAACAACTGCCCACCCAGTTGG
NJ1-1 (349)	TGGTGGGGTGGTCGCTCTTGCTGTCTTCCTCACGGAAACCCATCGCCGCCGCTCCCTCCTCGCGGAAACAACTGCCCACCCAGTTGG
NJ2-1 (349)	TGGTGGGGTGGTCGCTCTTGCTGTCTTCCTCACGGAAACCCATCGCCGCCGCTCCCTCCTCGCGGAAACAACTGCCCACCCAGTTGG
ZZYY3-1-2 (349)	TGGTGGGGTGGTCGCTCTTGCTGTCTTCCTCACGGAAACCCATCGCCGCCGCTCCCTCCTCGCGGAAACAACTGCCCACCCAGTTGG
CC2-1 (349)	TGGTGGGGTGGTCGCTCTTGCTGTCTTCCTCACGGAAACCCATCGCCGCCGCTCCCTCCTCGCGGAAACAACTGCCCACCCAGTTGG
CQ3-2 (349)	TGGTGGGGTGGTCGCTCTTGCTGTCTTCCTCACGGAAACCCATCGCCGCCGCTCCCTCCTCGCGGAAACAACTGCCCACCCAGTTGG
FJ6 (349)	TGGTGGGGTGGTCGCTCTTGCTGTCTTCCTCACGGAAACCCATCGCCGCCGCTCCCTCCTCGCGGAAACAACTGCCCACCCAGTTGG
FJ7-1 (349)	TGGTGGGGTGGTCGCTCTTGCTGTCTTCCTCACGGAAACCCATCGCCGCCGCTCCCTCCTCGCGGAAACAACTGCCCACCCAGTTGG
GD3-1 (349)	TGGTGGGGTGGTCGCTCTTGCTGTCTTCCTCACGGAAACCCATCGCCGCCGCTCCCTCCTCGCGGAAACAACTGCCCACCCAGTTGG
GZ4-1 (349)	TGGTGGGGTGGTCGCTCTTGCTGTCTTCCTCACGGAAACCCATCGCCGCCGCTCCCTCCTCGCGGRAACAACTGCCCACCCAGTTGG
GZ8-1 (349)	TGGTGGGGTGGTCGCTCTTGCTGTCTTCCTCACGGAAACCCATCGCCGCCGCTCCCTCCTCGCGGRAACAACTGCCCACCCAGTTGG
HB1-1 (349)	TGGTGGGGTGGTCGCTCTTGCTGTCTTCCTCACGGAAACCCATCGCCGCCGCTCCCTCCTCGCGGRAACAACTGCCCACCCAGTTGG
MA-1-1-1 (349)	TGGTGGGGTGGTCGCTCTTGCTGTCTTCCTCACGGAAACCCATCGCCGCCGCTCCCTCCTCGCGGAAACAACTGCCCACCCAGTTGG
MB-2-1-1 (349)	TGGTGGGGTGGTCGCTCTTGCTGTCTTCCTCACGGAAACCCATCGCCGCCGCTCCCTCCTCGCGGGAACAACTGCCCACCCAGTTGG
HN2-2 (349)	TGGTGGGGTGGTCGCTCTTGCTGTCTTCCTCACGGAAACCCATCGCCGCCGCTCCCTCCTCGCGGAAACAACTGCCCACCCAGTTGG
WJ1-1 (349)	TGGTGGGGTGGTCGCTCTTGCTGTCTTCCTCACGGAAACCCATCGCCGCCGCTCCCTCCTCGCGGAAACAACTGCCCACCCAGTTGG
ZZYY2-1-1 (349)	TGGTGGGGTGGTCGCTCTTGCTGTCTTCCTMACGGAAACCCATCGCCGCCGCTCCCTCCTCGCGGAAACAACTGCCCACCCAGTTGG
HZFY4-1-1 (349)	TGGTGGGGTGGTCGCTCTTGCTGTCTTCCTCACGGAAACCCATCGCCGCCGCTCCCTCCTCGCGGAAACAACTGCCCACCCAGTTGG
AG1IA-05500 (349)	TGGTGGGGTGGTCGCTCTTGCTGTCTTCCTCACGGAAACCCATCGCCGCCGCTCCCTCCTCGCGGAAACAACTGCCCACCCAGTTGG
AH3 (349)	TGGTGGGGTGGTCGCTCTTGCTGTCTTCCTCACGGAAACCCATCGCCGCCGCTCCCTCCTCGCGGAAACAACTGCCCACCCAGTTGG
HZFY5-2-1 (349)	TGGTGGGGTGGTCGCTCTTGCTGTCTTCCTCACGGAAACCCATCGCCGCCGCTCCCTCCTCGCGGAAACAACTGCCCACCCAGTTGG
FM1-1 (349)	TGGTGGGGTGGTCGCTCTTGCTGTCTTCCTCACGGAAACCCATCGCCGCCGCTCCCTCCTCGCGGAAACAACTGCCCACCCAGTTGG
FM2-1 (349)	TGGTGGGGTGGTCGCTCTTGCTGTCTTCCTCACGGAAACCCATCGCCGCCGCTCCCTCCTCGCGGAAACAACTGCCCACCCAGTTGG
SY1-1 (349)	TGGTGGGGTGGTCGCTCTTGCTGTCTTCCTCACGGAAACCCATCGCCGCCGCTCCCTCCTCGCGGAAACAACTGCCCACCCAGTTGG
SY5-1 (349)	TGGTGGGGTGGTCGCTCTTGCTGTCTTCCTCACGGAAACCCATCGCCGCCGCTCCCTCCTCGCGGAAACAACTGCCCACCCAGTTGG
WC2-1 (349)	TGGTGGGGTGGTCGCTCTTGCTGTCTTCCTCACGGAAACCCATCGCCGCCGCTCCCTCCTCGCGGAAACAACTGCCCACCCAGTTGG
Consensus (349)	TGGTGGGGTGGTCGCTCTTGCTGTCTTCCTCACGGAAACCCATCGCCGCCGCTCCCTCCTCGCGGAAACAACTGCCCACCCAGTTGG

Section 6

```
                (436) 436          450       460       470       480       490       500       510        522
          AG1IA (436) TACTGGCATGGACAACAAGGCTGCTGTGTACCTCAGCACCCTCCACCAACGCCTGTTCCGTCTTGCCCTCCCAACTGGGAGTGGAAC
          CC7-1 (436) TACTGGCATGGACAACAAGGCTGCTGTGTACCTCAGCACCCTCCACCAACGCCTGTTCCGTCTTGCCCTCCCAACTGGGAGTGGAAC
          NJ1-1 (436) TACTGGCATGGACAACAAGGCTGCTGTGTACCTCAGCACCCTCCACCAACGCCTGTTCCGTCTTGCCCTCCCAACTGGGAGTGGAAC
          NJ2-1 (436) TACTGGCATGGACAACAAGGCTGCTGTGTACCTCAGCACCCTCCACCAACGCCTGTTCCGTCTTGCCCTCCCAACTGGGAGTGGAAC
      ZZYY3-1-2 (436) TACTGGCATGGACAACAAGGCTGCTGTGTACCTCAGCACCCTCCACCAACGCCTGTTCCGTCTTGCCCTCCCAACTGGGAGTGGAAC
          CC2-1 (436) TACTGGCATGGACAACAAGGCTGCTGTGTACCTCAGCACCCTCCACCAACGCCTGTTCCGTCTTGCCCTCCCAACTGGGAGTGGAAC
          CQ3-2 (436) TACTGGCATGGACAACAAGGCTGCTGTGTACCTCAGCACCCTCCACCAACGCCTGTTCCGTCTTGCCCTCCCAACTGGGAGTGGAAC
            FJ6 (436) TACTGGCATGGACAACAAGGCTGCTGTGTACCTCAGCACCCTCCACCAACGCCTGTTCCGTCTTGCCCTCCCAACTGGGAGTGGAAC
          FJ7-1 (436) TACTGGCATGGACAACAAGGCTGCTGTGTACCTCAGCACCCTCCACCAACGCCTGTTCCGTCTTGCCCTCCCAACTGGGAGTGGAAC
          GD3-1 (436) TACTGGCATGGACAACAAGGCTGCTGTGTACCTCAGCACCCTCCACCAACGCCTGTTCCGTCTTGCCCTCCCAACTGGGAGTGGAAC
          GZ4-1 (436) TACTGGCATGGACAACAAGGCTGYTGTGTACCTCAGCACCCTCCACCAACGCCTGTTCCGTCTTGCCCTCCCAACTGGGAGTGGAAC
          GZ8-1 (436) TACTGGCATGGACAACAAGGCTGYTGTGTACCTCAGCACCCTCCACCAACGCCTGTTCCGTCTTGCCCTCCCAACTGGGAGTGGAAC
          HB1-1 (436) TACTGGCATGGACAACAAGGCTGYTGTGTACCTCAGCACCCTCCACCAACGCCTGTTCCGTCTTGCCCTCCCAACTGGGAGTGGAAC
       MA-1-1-1 (436) TACTGGCATGGACAACAAGGCTGYTGTGTACCTCAGCACCCTCCACCAACGCCTGTTCCGTCTTGCCCTCCCAACTGGGAGTGGAAC
       MB-2-1-1 (436) TACTGGCATGGACAACAAGGCTGTTGTGTACCTCAGCACCCTCCACCAACGCCTGTTCCGTCTTGCCCTCCCAACTGGGAGTGGAAC
          HN2-2 (436) TACTGGCATGGACAACAAGGCTGCTGTGTACCTCAGCACCCTCCACCAACGCCTGTTCCGTCTTGCCCTCCCAACTGGGAGTGGAAC
          WJ1-1 (436) TACTGGCATGGACAACAAGGCTGCTGTGTACCTCAGCACCCTCCACCAACGCCTGTTCCGTCTTGCCCTCCCAACTGGGAGTGGAAC
      ZZYY2-1-1 (436) TACTGGCATGGACAACAAGGCTGCTGTGTACCTCAGCACCCTCCACCAACGCCTGTTCCGTCTTGCCCTCCCAACTGGGAGTGGAAC
      HZFY4-1-1 (436) TACTGGCATGGACAACAAGGCTGCTGTGTACCTCAGCACCCTCCACCAACGCCTGTTCCGTCTTGCCCTCCCAACTGGGAGTGGAAC
    AG1IA-05500 (436) TACTGGCATGGACAACAAGGCTGCTGTGTACCTCAGCACCCTCCACCAACGCCTGTTCCGTCTTGCCCTCCCAACTGGGAGTGGAAC
            AH3 (436) TACTGGCATGGACAACAAGGCTGCTGTGTACCTCAGCACCCTCCACCAACGCCTGTTCCGTCTTGCCCTCCCAACTGGGAGTGGAAC
      HZFY5-2-1 (436) TACTGGCATGGACAACAAGGCTGCTGTGTACCTCAGCACCCTCCACCAACGCCTGTTCCGTCTTGCCCTCCCAACTGGGAGTGGAAC
          FM1-1 (436) TACTGGCATGGACAACAAGGCTGCTGTGTACCTCAGCACCCTCCACCAACGCCTGTTCCGTCTTGCCCTCCCAACTGGGAGTGGAAC
          FM2-1 (436) TACTGGCATGGACAACAAGGCTGCTGTGTACCTCAGCACCCTCCACCAACGCCTGTTCCGTCTTGCCCTCCCAACTGGGAGTGGAAC
          SY1-1 (436) TACTGGCATGGACAACAAGGCTGCTGTGTACCTCAGCACCCTCCACCAACGCCTGTTCCGTCTTGCCCTCCCAACTGGGAGTGGAAC
          SY5-1 (436) TACTGGCATGGACAACAAGGCTGCTGTGTACCTCAGCACCCTCCACCAACGCCTGTTCCGTCTTGCCCTCCCAACTGGGAGTGGAAC
          WC2-1 (436) TACTGGCATGGACAACAAGGCTGCTGTGTACCTCAGCACCCTCCACCAACGCCTGTTCCGTCTTGCCCTCCCAACTGGGAGTGGAAC
      Consensus (436) TACTGGCATGGACAACAAGGCTGCTGTGTACCTCAGCACCCTCCACCAACGCCTGTTCCGTCTTGCCCTCCCAACTGGGAGTGGAAC
```

Section 7

```
               (523) 523    530       540       550       560       570       580       590      609
          AG1IA (523) CCTTACACCCAATGCTGCAAGCCTACYCCTCCTACTCCACCTGGCCCCCAACCGAGCAAGCGCGCTACTACTGATACCAAGCGCCAC
          CC7-1 (523) CCTTACACCCAATGCTGCAAGCCTACYCCTCCTACTCCACCTGGCCCCCAACCGAGCAAGCGCGCTACTACTGATACCAAGCGCCAC
          NJ1-1 (523) CCTTACACCCAATGCTGCAAGCCTACYCCTCCTACTCCACCTGGCCCCCAACCGAGCAAGCGCGCTACTACTGATACCAAGCGCCAC
          NJ2-1 (523) CCTTACACCCAATGCTGCAAGCCTACYCCTCCTACTCCACCTGGCCCCCAACCGAGCAAGCGCGCTACTACTGATACCAAGCGCCAC
      ZZYY3-1-2 (523) CCTTACACCCAATGCTGCAAGCCTACYCCTCCTACTCCACCTGGCCCCCAACCGAGCAAGCGCGCTACTACTGATACCAAGCGCCAC
          CC2-1 (523) CCTTACACCCAATGCTGCAAGCCTACCCCTCCTACTCCACCTGGCCCCCAACCGAGCAAGCGCGCTACTACTGATACCAAGCGCCAC
          CQ3-2 (523) CCTTACACCCAATGCTGCAAGCCTACCCCTCCTACYCCACCTGGCCCCCAACCGAGCAAGCGCGCTACTACTGATACCAAGCGCCAC
            FJ6 (523) CCTTACACCCAATGCTGCAAGCCTACCCCTCCTACTCCACCTGGCCCCCAACCGAGCAAGCGCGCTACTACTGATACCAAGCGCCAC
          FJ7-1 (523) CCTTACACCCAATGCTGCAAGCCTACCCCTCCTACTCCACCTGGCCCCCAACCGAGCAAGCGCGCTACTACTGATACCAAGCGCCAC
          GD3-1 (523) CCTTACACCCAATGCTGCAAGCCTACCCCTCCTACYCCACCTGGCCCCCAACCGAGCAAGCGCGCTACTACTGATACCAAGCGCCAC
          GZ4-1 (523) CCTTACACCCAATGCTGCAAGCCTACCCCYCCTACTCCACCYGGCCCCCAACCGAGCAAGCGCGCTACTASTGAKACCAAGCGCCAC
          GZ8-1 (523) CCTTACACCCAATGCTGCAAGCCTACCCCYCCTACTCCACCYGGCCCCCAACCGAGCAAGCGCGCTACTASTGAKACCAAGCGCCAC
          HB1-1 (523) CCTTACACCCAATGCTGCAAGCCTACCCCYCCTACTCCACCYGGCCCCCAACCGAGCAAGCGCGCTACTASTGAKACCAAGCGCCAC
       MA-1-1-1 (523) CCTTACACCCAATGCTGCAAGCCTACCCCYCCTACYCCACCYGGCCCCCAACCGAGCAAGCGCGCTACTASTGAKACCAAGCGCCAC
       MB-2-1-1 (523) CCTTACACCCAATGCTGCAAGCCTACCCCCCCTACTCCACCCGGCCCCCAACCGAGCAAGCGCGCTACTAGTGAGACCAAGCGCCAC
          HN2-2 (523) CCTTACACCCAATGCTGCAAGCCTACTCCTCCTACTCCACCTGGCCCCCAACCGAGCAAGCGCGCTACTACTGATACCAAGCGCCAC
          WJ1-1 (523) CCTTACACCCAATGCTGCAAGCCTACYCCTCCTACTCCACCTGGCCCCCAACCGAGCAAGCGCGCTACTACTGATACCAAGCGCCAC
      ZZYY2-1-1 (523) CCTTACACCCAATGCTGCAAGCCTACYCCTCCTACTCCACCTGGCCCCCAACCGAGCAAGCGCGCTACTACTGATACCAAGCGCCAC
      HZFY4-1-1 (523) CCTTACACCCAATGCTGCAAGCCTACCCCTCCTACTCCACCTGGCCCCCAACCGAGCAAGCGCGCTACTACTGATACCAAGCGCCAC
    AG1IA-05500 (523) CCTTACACCCAATGCTGCAAGCCTACTCCTCCTACTCCACCTGGCCCCCAACCGAGCAAGCGCGCTACTACTGATACCAAGCGCCAC
            AH3 (523) CCTTACACCCAATGCTGCAAGCCTACCCCTCCTACTCCACCTGGCCCCCAACCGAGCAAGCGCGCTACTACTGATACCAAGCGCCAC
      HZFY5-2-1 (523) CCTTACACCCAATGCTGCAAGCCTACCCCTCCTACTCCACCTGGCCCCCAACCGAGCAAGCGCGCTACTACTGATACCAAGCGCCAC
          FM1-1 (523) CCTTACACCCAATGCTGCAAGCCTACCCCTCCTACTCCACCTGGCCCCCAACCGAGCAAGCGCGCTACTACTGATACCAAGCGCCAC
          FM2-1 (523) CCTTACACCCAATGCTGCAAGCCTACCCCTCCTACTCCACCTGGCCCCCAACCGAGCAAGCGCGCTACTACTGATACCAAGCGCCAC
          SY1-1 (523) CCTTACACCCAATGCTGCAAGCCTACCCCTCCTACTCCACCTGGCCCCCAACCGAGCAAGCGCGCTACTACTGATACCAAGCGCCAC
          SY5-1 (523) CCTTACACCCAATGCTGCAAGCCTACCCCTCCTACTCCACCTGGCCCCCAACCGAGCAAGCGCGCTACTACTGATACCAAGCGCCAC
          WC2-1 (523) CCTTACACCCAATGCTGCAAGCCTACCCCTCCTACTCCACCTGGCCCCCAACCGAGCAAGCGCGCTACTACYGATACCAAGCGCCAC
      Consensus (523) CCTTACACCCAATGCTGCAAGCCTACCCCTCCTACTCCACCTGGCCCCCAACCGAGCAAGCGCGCTACTACTGATACCAAGCGCCAC
```

Section 8

```
              (610) 610       620       630       640       650       660       670       680      696
        AG1IA (610) AATGCTGTCCCTCAGCACAAGAAGAAGGCGACTGCCCAGTACACCGAGCTCGAGGCTTTCTTCTGCCCCGCGGGCATGACCGCTTGC
        CC7-1 (610) AATGCTGTCCCTCAGCACAAGAAGAAGGCGACTGCCCAGTACACCGAGCTCGAGGCTTTCTTCTGCCCCGCGGGCATGACCGCTTGC
        NJ1-1 (610) AATGCTGTCCCTCAGCACAAGAAGAAGGCGACTGCCCAGTACACCGAGCTCGAGGCTTTCTTCTGCCCCGCGGGCATGACCGCTTGC
        NJ2-1 (610) AATGCTGTCCCTCAGCACAAGAAGAAGGCGACTGCCCAGTACACCGAGCTCGAGGCTTTCTTCTGCCCCGCGGGCATGACCGCTTGC
    ZZYY3-1-2 (610) AATGCTGTCCCTCAGCACAAGAAGAAGGCGACTGCCCAGTACACCGAGCTCGAGGCTTTCTTCTGCCCCGCGGGCATGACCGCTTGC
        CC2-1 (610) AATGCTGTCCCTCAGCACAAGAAGAAGGCGACTGCCCAGTACACCGAGCTCGAGGCTTTCTTCTGCCCCGCGGGCATGACCGCTTGC
         CQ3-2 (610) AATGCTGTCCCTCAGCACAAGAAGAAGGCGACTGCYCAGTACACCGAGCTCGAGGCTTTCTTCTGCCCCGCGGGCATGACCGCTTGC
          FJ6 (610) AATGCTGTCCCTCAGCACAAGAAGAAGGCGACTGCYCAGTACACCGAGCTCGAGGCTTTCTTCTGCCCCGCGGGCATGACCGCTTGC
        FJ7-1 (610) AATGCTGTCCCTCAGCACAAGAAGAAGGCGACTGCYCAGTACACCGAGCTCGAGGCTTTCTTCTGCCCCGCGGGCATGACCGCTTGC
        GD3-1 (610) AATGCTGTCCCTCAGCACAAGAAGAAGGCGACTGCTCAGTACACCGAGCTCGAGGCTTTCTTCTGCCCCGCGGGCATGACCGCTTGC
        GZ4-1 (610) AATGCTGTCCCTCAGCACAAGAAGAAGGCRACTGCYCAGTACACCGAGCTCGAGGCTTTCTTCTGCCCCGCGGGCATGACCGCTTGC
        GZ8-1 (610) AATGCTGTCCCTCAGCACAAGAAGAAGGCRACTGCYCAGTACACCGAGCTCGAGGCTTTCTTCTGCCCCGCGGGCATGACCGCTTGC
        HB1-1 (610) AATGCTGTCCCTCAGCACAAGAAGAAGGCRACTGCTCAGTACACCGAGCTCGAGGCTTTCTTCTGCCCCGCGGGCATGACCGCTTGC
     MA-1-1-1 (610) AATGCTGTCCCTCAGCACAAGAAGAAGGCGACTGCTCAGTACACCGARCTCGAGGCTTTCTTCTGCCCCGCGGGCATGACCGCTTGC
     MB-2-1-1 (610) AATGCTGTCCCTCAGCACAAGAAGAAGGCAACTGCTCAGTACACCGAGCTCGAGGCTTTCTTCTGCCCCGCGGGCATGACCGCTTGC
        HN2-2 (610) AATGCTGTCCCTCAGCACAAGAAGAAGGCGACTGCCCAGTACACCGAGCTCGAGGCTTTCTTCTGCCCCGCGGGCATGACCGCTTGC
        WJ1-1 (610) AATGCTGTCCCTCAGCACAAGAAGAAGGCGACTGCCCAGTACACCGAGCTCGAGGCTTTCTTCTGCCCCGCGGGCATGACCGCTTGC
    ZZYY2-1-1 (610) AATGCTGTCCCTCAGCACAAGAAGAAGGCGACTGCCCAGTACACCGAGCTCGAGGCTTTCTTCTGCCCCGCGGGCATGACCGCTTGC
    HZFY4-1-1 (610) AATGCTGTCCCTCAGCACAAGAAGAAGGCGACTGCCCAGTACACCGAGCTCGAGGCTTTCTTCTGCCCCGCGGGCATGACCGCTTGC
  AG1IA-05500 (610) AATGCTGTCCCTCAGCACAAGAAGAAGGCGACTGCCCAGTACACCGAGCTCGAGGCTTTCTTCTGCCCCGCGGGCATGACCGCTTGC
          AH3 (610) AATGCTGTCCCTCAGCACAAGAAGAAGGCGACTGCYCAGTACACCGAGCTCGAGGCTTTCTTCTGCCCCGCGGGCATGACCGCTTGC
    HZFY5-2-1 (610) AATGCTGTCCCTCAGCACAAGAAGAAGGCGACTGCYCAGTACACCGAGCTCGAGGCTTTCTTCTGCCCCGCGGGCATGACCGCTTGC
        FM1-1 (610) AATGCTGTCCCTCAGCACAAGAAGAAGGCGACTGCCCAGTACACCGAGCTCGAGGCTTTCTTCTGCCCCGCGGGCATGACCGCTTGC
        FM2-1 (610) AATGCTGTCCCTCAGCACAAGAAGAAGGCGACTGCCCAGTACACCGAGCTCGAGGCTTTCTTCTGCCCCGCGGGCATGACCGCTTGC
        SY1-1 (610) AATGCTGTCCCTCAGCACAAGAAGAAGGCGACTGCCCAGTACACCGAGCTCGAGGCTTTCTTCTGCCCCGCGGGCATGACCGCTTGC
        SY5-1 (610) AATGCTGTCCCTCAGCACAAGAAGAAGGCGACTGCCCAGTACACCGAGCTCGAGGCTTTCTTCTGCCCCGCGGGCATGACCGCTTGC
        WC2-1 (610) AATGCTGTCCCTCAGCACAAGAAGAAGGCGACTGCCCAGTACACCGAGCTCGAGGCTTTCTTCTGCCCCGCGGGCATGACCGCTTGC
    Consensus (610) AATGCTGTCCCTCAGCACAAGAAGAAGGCGACTGCCCAGTACACCGAGCTCGAGGCTTTCTTCTGCCCCGCGGGCATGACCGCTTGC
```

Section 9

```
           (697) 697          710       720       730       740       750       760       770          783
       AG1IA (697) CCTATCGGCGGGATCTTCAGCACCGAGTCTGAATGCTTCGATACGCTTTCCGACCTGAAATCTTGCGGAGGTTGCGCTTCGGTGGGC
       CC7-1 (697) CCTATCGGCGGGATCTTCAGCACCGAGTCTGAATGCTTCGATACGCTTTCCGACCTGAAATCTTGCGGAGGTTGCGCTTCGGTGGGC
       NJ1-1 (697) CCTATCGGCGGGATCTTCAGCACCGAGTCTGAATGCTTCGATACGCTTTCCGACCTGAAATCTTGCGGAGGTTGCGCTTCGGTGGGC
       NJ2-1 (697) CCTATCGGCGGGATCTTCAGCACCGAGTCTGAATGCTTCGATACGCTTTCCGACCTGAAATCTTGCGGAGGTTGCGCTTCGGTGGGC
   ZZYY3-1-2 (697) CCTATCGGCGGGATCTTCAGCACCGAGTCTGAATGCTTCGATACGCTTTCCGACCTGAAATCTTGCGGAGGTTGCGCTTCGGTGGGC
       CC2-1 (697) CCTATCGGCGGGATCTTCAGCACCGAGTCTGAATGCTTCGATACGCTTTCCGACCTGAAATCTTGCGGAGGTTGCGCTTCGGTGGGC
       CQ3-2 (697) CCTATCGGCGGGATCTTCAGCACCGAGTCTGAATGCTTCGATACGCTTTCCGACCTGAAATCTTGCGGAGGTTGCGCTTCGGTGGGC
         FJ6 (697) CCTATCGGCGGGATCTTCAGCACCGAGTCTGAATGCTTCGATACGCTTTCCGACCTGAAATCTTGCGGAGGTTGCGCTTCGGTGGGC
       FJ7-1 (697) CCTATCGGCGGGATCTTCAGCACCGAGTCTGAATGCTTCGATACGCTTTCCGACCTGAAATCTTGCGGAGGTTGCGCTTCGGTGGGC
       GD3-1 (697) CCTATCGGCGGGATCTTCAGCACCGAGTCTGAATGCTTCGATACGCTTTCCGACCTGAAATCTTGCGGAGGTTGCGCTTCGGTGGGC
       GZ4-1 (697) CCTATCGGSGGRATCTTCAGCACCGAGTCTGAATGCTTCGATACGCTTTCCGACCTGAAATCTTGCGGAGGTTGCGCTTCGGTGGGC
       GZ8-1 (697) CCTATCGGSGGRATCTTCAGCACCGAGTCTGAATGCTTCGATACGCTTTCCGACCTGAAATCTTGCGGAGGTTGCGCTTCGGTGGGC
       HB1-1 (697) CCTATCGGSGGRATCTTCAGCACCGAGTCTGAATGCTTCGATACGCTTTCCGACCTGAAATCTTGCGGAGGTTGCGCTTCGGTGGGC
     MA-1-1-1 (697) CCTATCGGCGGGATCTTCAGCACCGAGTCTGAATGCTTCGATACGCTTTCCGACCTGAAATCTTGCGGAGGTTGCGCTTCGGTGGGC
     MB-2-1-1 (697) CCTATCGGGGGAATCTTCAGCACCGAGTCTGAATGCTTCGATACGCTTTCCGACCTGAAATCTTGCGGAGGTTGCGCTTCGGTGGGC
       HN2-2 (697) CCTATCGGCGGGATCTTCAGCACCGAGTCTGAATGCTTCGATACGCTTTCCGACCTGAAATCTTGCGGAGGTTGCGCTTCGGTGGGC
       WJ1-1 (697) CCTATCGGCGGGATCTTCAGCACCGAGTCTGAATGCTTCGATACGCTTTCCGACCTGAAATCTTGCGGAGGTTGCGCTTCGGTGGGC
   ZZYY2-1-1 (697) CCTATCGGCGGGATCTTCAGCACCGAGTCTGAATGCTTCGATACGCTTTCCGACCTGAAATCTTGCGGAGGTTGCGCTTCGGTGGGC
    HZFY4-1-1 (697) CCTATCGGCGGGATCTTCAGCACCGAGTCTGAATGCTTCGATACGCTTTCCGACCTGAAATCTTGCGGAGGTTGCGCTTCGGTGGGC
  AG1IA-05500 (697) CCTATCGGCGGGATCTTCAGCACCGAGTCTGAATGCTTCGATACGCTTTCCGACCTGAAATCTTGCGGAGGTTGCGCTTCGGTGGGC
         AH3 (697) CCTATCGGCGGGATCTTCAGCACCGAGTCTGAATGCTTCGATACGCTTTCCGACCTGAAATCTTGCGGAGGTTGCGCTTCGGTGGGC
    HZFY5-2-1 (697) CCTATCGGCGGGATCTTCAGCACCGAGTCTGAATGCTTCGATACGCTTTCCGACCTGAAATCTTGCGGAGGTTGCGCTTCGGTGGGC
       FM1-1 (697) CCTATCGGCGGGATCTTCAGCACCGAGTCTGAATGCTTCGATACGCTTTCCGACCTGAAATCTTGCGGAGGTTGCGCTTCGGTGGGC
       FM2-1 (697) CCTATCGGCGGGATCTTCAGCACCGAGTCTGAATGCTTCGATACGCTTTCCGACCTGAAATCTTGCGGAGGTTGCGCTTCGGTGGGC
       SY1-1 (697) CCTATCGGCGGGATCTTCAGCACCGAGTCTGAATGCTTCGATACGCTTTCCGACCTGAAATCTTGCGGAGGTTGCGCTTCGGTGGGC
       SY5-1 (697) CCTATCGGCGGGATCTTCAGCACCGAGTCTGAATGCTTCGATACGCTTTCCGACCTGAAATCTTGCGGAGGTTGCGCTTCGGTGGGC
       WC2-1 (697) CCTATCGGCGGGATCTTCAGCACCGAGTCTGAATGCTTCGATACGCTTTCCGACCTGAAATCTTGCGGAGGTTGCGCTTCGGTGGGC
   Consensus (697) CCTATCGGCGGGATCTTCAGCACCGAGTCTGAATGCTTCGATACGCTTTCCGACCTGAAATCTTGCGGAGGTTGCGCTTCGGTGGGC
```

Section 10

```
              (784) 784   790       800       810       820       830       840       850       860       870
        AG1IA (784) GCTGGACAAGATTGCTCTGCGATCCCCAACGTTAGCGTCTCGTCGTGCGAGAGCGGCGGTTGCACTGTCTTGTCTTGCAACGAAGGT
        CC7-1 (784) GCTGGACAAGATTGCTCTGCGATCCCCAACGTTAGCGTCTCGTCGTGCGAGAGCGGCGGTTGCACTGTCTTGTCTTGCAACGAAGGT
        NJ1-1 (784) GCTGGACAAGATTGCTCTGCGATCCCCAACGTTAGCGTCTCGTCGTGCGAGAGCGGCGGTTGCACTGTCTTGTCTTGCAACGAAGGT
        NJ2-1 (784) GCTGGACAAGATTGCTCTGCGATCCCCAACGTTAGCGTCTCGTCGTGCGAGAGCGGCGGTTGCACTGTCTTGTCTTGCAACGAAGGT
    ZZYY3-1-2 (784) GCTGGACAAGATTGCTCTGCGATCCCCAACGTTAGCGTCTCGTCGTGCGAGAGCGGCGGTTGCACTGTCTTGTCTTGCAACGAAGGT
        CC2-1 (784) GCTGGACAAGATTGCTCTGCGATCCCCAACGTTAGCGTCTCGTCGTGCGAGAGCGGCGGTTGCACTGTCTTGTCTTGCAACGAAGGT
        CQ3-2 (784) GCTGGACAAGATTGCTCTGCGATCCCCAACGTTAGCGTCTCGTCGTGCGAGAGCGGCGGTTGCACTGTCTTGTCTTGCAACGAAGGT
          FJ6 (784) GCTGGACAAGATTGCTCTGCGATCCCCAACGTTAGCGTCTCGTCRTGCGAGAGCGGCGGTTGYACTGTCTTGTCTTGCAACGAAGGT
        FJ7-1 (784) GCTGGACAAGATTGCTCTGCGATCCCCAACGTTAGCGTCTCGTCRTGCGAGAGCGGCGGTTGYACTGTCTTGTCTTGCAACGAAGGT
        GD3-1 (784) GCTGGACAAGATTGCTCTGCGATCCCCAACGTTAGCGTCTCGTCRTGCGAGAGCGGCGGTTGYACTGTCTTGTCTTGCAACGAAGGT
        GZ4-1 (784) GCTGGRCAAGATTGCTCTGCGATCCCCAACGTTAGCGTCTCGTCGTGCGAGAGCGGCGGTTGCWCTGTCTTGTCTTGCAACGAAGGT
        GZ8-1 (784) GCTGGRCAAGATTGCTCTGCGATCCCCAACGTTAGCGTCTCGTCGTGCGAGAGCGGCGGTTGCWCTGTCTTGTCTTGCAACGAAGGT
        HB1-1 (784) GCTGGRCAAGATTGCTCTGCGATCCCCAACGTTAGCGTCTCGTCGTGCGAGAGCGGCGGTTGCWCTGTCTTGTCTTGCAACGAAGGT
      MA-1-1-1 (784) GCTGGACAAGATTGCTCTGCGATCCCCAACGTTAGCGTCTCGTCGTGCGAGAGCGGCGGTTGCACTGTCTTGTCTTGCAACGAAGGT
      MB-2-1-1 (784) GCTGGRCAAGATTGCTCTGCGATCCCCAACGTTAGCGTCTCGTCGTGCGAGAGCGGCGGTTGYACTGTCTTGTCTTGCAACGAAGGT
        HN2-2 (784) GCTGGACAAGATTGCTCTGCGATCCCCAACGTTAGCGTCTCGTCGTGCGAGAGCGGCGGTTGCACTGTCTTGTCTTGCAACGAAGGT
        WJ1-1 (784) GCTGGACAAGATTGCTCTGCGATCCCCAACGTTAGCGTCTCGTCGTGCGAGAGCGGCGGTTGCACTGTCTTGTCTTGCAACGAAGGT
    ZZYY2-1-1 (784) GCTGGACAAGATTGCTCTGCGATCCCCAACGTTAGCGTCTCGTCGTGCGAGAGCGGCGGTTGCACTGTCTTGTCTTGCAACGAAGGT
    HZFY4-1-1 (784) GCTGGACAAGATTGCTCTGCGATCCCCAACGTTAGCGTCTCGTCGTGCGAGAGCGGCGGTTGCACTGTCTTGTCTTGCAACGAAGGT
  AG1IA-05500 (784) GCTGGACAAGATTGCTCTGCGATCCCCAACGTTAGCGTCTCGTCGTGCGAGAGCGGCGGTTGCACTGTCTTGTCTTGCAACGAAGGT
          AH3 (784) GCTGGACAAGATTGCTCTGCGATCCCCAACGTTAGCGTCTCGTCGTGCGAGAGCGGCGGTTGCACTGTCTTGTCTTGCAACGAAGGT
    HZFY5-2-1 (784) GCTGGACAAGATTGCTCTGCGATCCCCAACGTTAGCGTCTCGTCGTGCGAGAGCGGCGGTTGCACTGTCTTGTCTTGCAACGAAGGT
        FM1-1 (784) GCTGGACAAGATTGCTCTGCGATCCCCAACGTTAGCGTCTCGTCGTGCGAGAGCGGCGGTTGCACTGTCTTGTCTTGCAACGAAGGT
        FM2-1 (784) GCTGGACAAGATTGCTCTGCGATCCCCAACGTTAGCGTCTCGTCGTGCGAGAGCGGCGGTTGCACTGTCTTGTCTTGCAACGAAGGT
        SY1-1 (784) GCTGGACAAGATTGCTCTGCGATCCCCAACGTTAGCGTCTCGTCGTGCGAGAGCGGCGGTTGCACTGTCTTGTCTTGCAACGAAGGT
        SY5-1 (784) GCTGGACAAGATTGCTCTGCGATCCCCAACGTTAGCGTCTCGTCGTGCGAGAGCGGCGGTTGCACTGTCTTGTCTTGCAACGAAGGT
        WC2-1 (784) GCTGGACAAGATTGCTCTGCGATCCCCAACGTTAGCGTCTCGTCGTGCGAGAGCGGCGGTTGCACTGTCTTGTCTTGCAACGAAGGT
    Consensus (784) GCTGGACAAGATTGCTCTGCGATCCCCAACGTTAGCGTCTCGTCGTGCGAGAGCGGCGGTTGCACTGTCTTGTCTTGCAACGAAGGT
```

```
            (871) 871      880       890       900        912
      AG1IA (871) TATACCGTTAGCGAAGACAAACAATCGTGCGTTGCCATCTAA
      CC7-1 (871) TATACCGTTAGCGAAGACAAACAATCGTGCGTTGCCATCTAA
      NJ1-1 (871) TATACCGTTAGCGAAGACAAACAATCGTGCGTTGCCATCTAA
      NJ2-1 (871) TATACCGTTAGCGAAGACAAACAATCGTGCGTTGCCATCTAA
   ZZYY3-1-2 (871) TATACCGTTAGCGAAGACAAACAATCGTGCGTTGCCATCTAA
      CC2-1 (871) TATACCGTTAGCGAAGACAAACAATCGTGCGTTGCCATCTAA
      CQ3-2 (871) TATACCGTTAGCGAAGACAAACAATCGTGCGTTGCCATCTAA
        FJ6 (871) TATACCGTTAGCGAAGACAAACAATCGTGCGTTGCCATCTAA
      FJ7-1 (871) TATACCGTTAGCGAAGACAAACAATCGTGCGTTGCCATCTAA
      GD3-1 (871) TATACCGTTAGCGAAGACAAACAATCGTGCGTTGCCATCTAA
      GZ4-1 (871) TATACCGTTAGCGAAGACAAACAATCGTGCGTTGCCATCTAA
      GZ8-1 (871) TATACCGTTAGCGAAGACAAACAATCGTGCGTTGCCATCTAA
      HB1-1 (871) TATACCRTTAGCGAAGACAAACAATCGTGCGTTGCCATCTAA
    MA-1-1-1 (871) TATACCRTTAGCGAAGACAAACAATCGTGCGTTGCCATCTAA
    MB-2-1-1 (871) TATACCGTTAGCGAAGACAAACAATCGTGCGTTGCCATCTAA
      HN2-2 (871) TATACCGTTAGCGAAGACAAACAATCGTGCGTTGCCATCTAA
      WJ1-1 (871) TATACCGTTAGCGAAGACAAACAATCGTGCGTTGCCATCTAA
   ZZYY2-1-1 (871) TATACCGTTAGCGAAGACAAACAATCGTGCGTTGCCATCTAA
   HZFY4-1-1 (871) TATACCGTTAGCGAAGACAAACAATCGTGCGTTGCCATCTAA
 AG1IA-05500 (871) TATACCGTTAGCGAAGACAAACAATCGTGCGTTGCCATCTAA
        AH3 (871) TATACCGTTAGCGAAGACAAACAATCGTGCGTTGCCATCTAA
   HZFY5-2-1 (871) TATACCRTTAGCGAAGACAAACAATCGTGCGTTGCCATCTAA
      FM1-1 (871) TATACCGTTAGCGAAGACAAACAATCGTGCGTTGCCATCTAA
      FM2-1 (871) TATACCGTTAGCGAAGACAAACAATCGTGCGTTGCCATCTAA
      SY1-1 (871) TATACCGTTAGCGAAGACAAACAATCGTGCGTTGCCATCTAA
      SY5-1 (871) TATACCGTTAGCGAAGACAAACAATCGTGCGTTGCCATCTAA
      WC2-1 (871) TATACCGTTAGCGAAGACAAACAATCGTGCGTTGCCATCTAA
   Consensus (871) TATACCGTTAGCGAAGACAAACAATCGTGCGTTGCCATCTAA
```
